# Coexistence of the Perfluorinated Cp* Anion With Oxidizing and Electrophilic Cations

**DOI:** 10.1002/chem.202500743

**Published:** 2025-04-17

**Authors:** Robin Sievers, Nico G. Kub, Tim‐Niclas Streit, Susanne M. Rupf, Moritz Malischewski

**Affiliations:** ^1^ Institute of Chemistry and Biochemistry – Inorganic Chemistry Freie Universität Berlin Fabeckstr. 34/36 14195 Berlin Germany

**Keywords:** anions, carbanions, cations, cyclopentadienyl ligands, fluorinated ligands

## Abstract

The air‐ and water‐stable perfluorinated Cp* [C_5_(CF_3_)_5_]^−^ is presented as a candidate for the vastly underexplored group of weakly coordinating carbanions (WCCAs). Its extreme electron deficiency, combined with the stabilization of the negative charge within an aromatic system, results in a low basicity, yielding a very weak coordination ability. As Cp anions usually possess a strongly pronounced carbanionic character, the perfluorinated Cp* resembles an extraordinary exception for both the WCA and Cp chemistry. However, the coordination ability remains ambivalent due to the substitution lability of many of its complexes, allowing for the formation of unique ligand‐WCA switches. Due to the low reactivity, there is a need for new transfer reagents containing the perfluorinated Cp* in combination with reactive cations. Thus, we report the synthesis and complete characterization of [C_5_(CF_3_)_5_]^−^ salts with hydride‐accepting [(C_6_H_5_)_3_C]^+^, valuable Ag(I) reagents, oxidizing [Fe(C_5_H_5_)_2_]^+^ or [N(*p*‐C_6_H_4_Br)_3_]^+^ and Brønsted acidic [H(*m,m*‐NC_5_H_3_F_2_)_2_]^+^. Notably, these unprecedented ion pairs are exclusively accessible and stabilized by the low coordination affinity and pronounced oxidative resistance of the perfluorinated Cp*.

## Graphical abstract

The perfluorinated Cp* [C_5_(CF_3_)_5_]^−^ is known for its unique ligand/counteranion switchability and is now introduced as a weakly coordinating anion (WCA). Its extraordinarily low nucleophilicity, combined with its pronounced oxidative stability, allows for the coexistence with reactive cations and the formation of unprecedented salts.

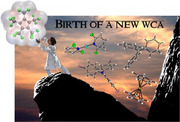

## 1 Introduction

As preparative and analytical capabilities have evolved in recent years, the hunt for labile and reactive cations has become an avid pursuit for many chemists. This naturally leads to an increasing demand for weakly coordinating anions (WCAs),^[^ ^1^, ^2^, ^3^, ^4^ ^]^ which, in addition to their general accessibility, are required to exhibit pronounced stability against electrophiles or oxidation and minimal coordination ability towards their respective cations.^[^ ^2^ ^]^ This is mainly achieved by large monoanions with a diffuse charge delocalization. While simple binary WCAs (e.g., [ClO_4_]^−^, [BF_4_]^−^, [MF_6_]^−^ (M = Sb, As)) still enjoy great popularity,^[^ ^5^, ^6^, ^7^, ^8^, ^9^, ^10^, ^11^, ^12^ ^]^ the trend is towards larger tailored anions. Famous examples are carborates,^[^ ^13^, ^14^, ^15^ ^]^ borates with highly fluorinated substituents, such as [BAr^F^_4_]^−^ (Ar^F^ = C_6_F_5_, 3,5‐C_6_H_3_(CF_3_)_2_),^[^ ^16^, ^17^ ^]^ teflate‐based [M(OTeF_5_)_4_]^−^ (M = B, Al, Ga)^[^ ^18^, ^19^, ^20^, ^21^, ^22^ ^]^ or [M(OTeF_5_)_6_]^−^ (M = Sb, As)^[^ ^14^, ^23^, ^24^ ^]^ and the extremely weak coordinating alkoxyaluminate [Al(OR^F^)_4_]^−^ (R^F^ = C(CF_3_)_3_) of the Krossing group.^[^ ^25^, ^26^, ^27^, ^28^, ^29^ ^]^ However, the vast majority of WCAs are based on triels and pnictogens,^[^ ^30^, ^31^, ^32^, ^33^, ^34^, ^35^, ^36^, ^37^, ^38^, ^39^ ^]^ leaving the group of tetrels in between almost untouched.^[^ ^40^ ^]^ This is mainly due to the fact that the anions of the tetrel elements, and especially their respective carbanions, are typically considered to be reactive and highly basic species.

**Figure 1:**
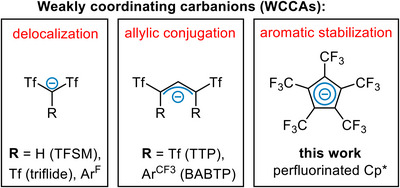
Examples of WCCAs, categorized by their negative charge delocalization within the carbon‐system. Tf = SO_2_CF_3_, Ar^F ^= C_6_F_5_, Ar^CF3 ^= 3,5‐C_6_H_3_(CF_3_)_2_.

Thus, these weakly coordinating carbanions (WCCAs) represent a rare and highly unexplored domain within the field of WCAs. The stabilization of the negative charge is mainly achieved by strong electron withdrawing substituents (e.g., SO_2_CF_3_ and C_6_F_5_) as demonstrated by TFSM (bis(triflyl)methanide),^[^ ^41^, ^42^ ^]^ triflide^[^ ^43^ ^]^ and its aryl substituted analog by Yamamoto (Figure 1, left).^[^ ^44^, ^45^ ^]^ Seminal works by List and Gessner extended these findings with an additional negative charge delocalization by conjugation within an allylic system in TTP (1,1,3,3‐tetratriflylpropenyl)^[^ ^46^ ^]^ and BABTP (1,3‐bis(3,5‐bis(trifluoromethylphenyl)‐1,3‐bis(triflyl)propenyl)^[^ ^47^, ^48^ ^]^ respectively (Figure 1, middle). While the protonated forms of the common weakly coordinating anions [H][WCA] potentially offer strong reactivities, they suffer from stability problems due to pronounced protonolysis.^[^ ^13^, ^23^, ^49^ ^]^ Advantageously, C‐H acidic [H][WCCAs] are rather stable and suitable for synthetically useful introduction reactions for labile cations. Moreover, they can act as potent Brønsted acid catalysts, as demonstrated by List's group with their [H][TTP] initiated organocatalysis.^[^ ^50^ ^]^

**Scheme 1:**
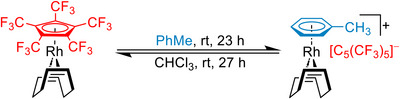
Exemplary quantitative and reversible substitution of the perfluorinated Cp* in [Rh(COD)(C_5_(CF_3_)_5_)] by toluene.

Recently, we introduced the perfluorinated Cp* [C_5_(CF_3_)_5_]^−^ to coordination chemistry and now propose its suitability for the group of WCCAs (Figure 1, right).^[^ ^51^, ^52^ ^]^ The combination of strongly electron withdrawing CF_3_‐groups together with the negative charge delocalization in an aromatic system generally results in a very low coordination ability. However, while simple WCAs such as [BF_4_]^−^ or [SbF_6_]^−^ tend to coordinate to strongly Lewis acidic metal centers, and tailored carborates or Krossing's alkoxyaluminates have been designed to be the least coordinating, the perfluorinated Cp* exhibits an ambivalent coordination ability. This was remarkably demonstrated by its fully reversible ligand‐WCA switchability, representing a unique reactivity within organometallic chemistry (Scheme 1).^[^ ^53^, ^54^, ^55^ ^]^ As Cp anions usually possess a pronounced carbanionic character with a strong affinity for E‐C bond formation throughout the whole periodic table, the perfluorinated Cp* represents an unprecedented exception from this for the class of WCAs. Therefore, it was aimed to synthesize unseen ion pairs, consisting of [C_5_(CF_3_)_5_]^−^ as a weakly coordinating anion. The utilized reactive cations have strong oxidizing and electrophilic properties, emphasizing the extremely low nucleophilicity and high oxidative resistance of the perfluorinated Cp*. Furthermore, the prepared salts also provide a basis of valuable transfer reagents for future synthetic applications.

## 2 Results and Discussion

**Figure 2:**
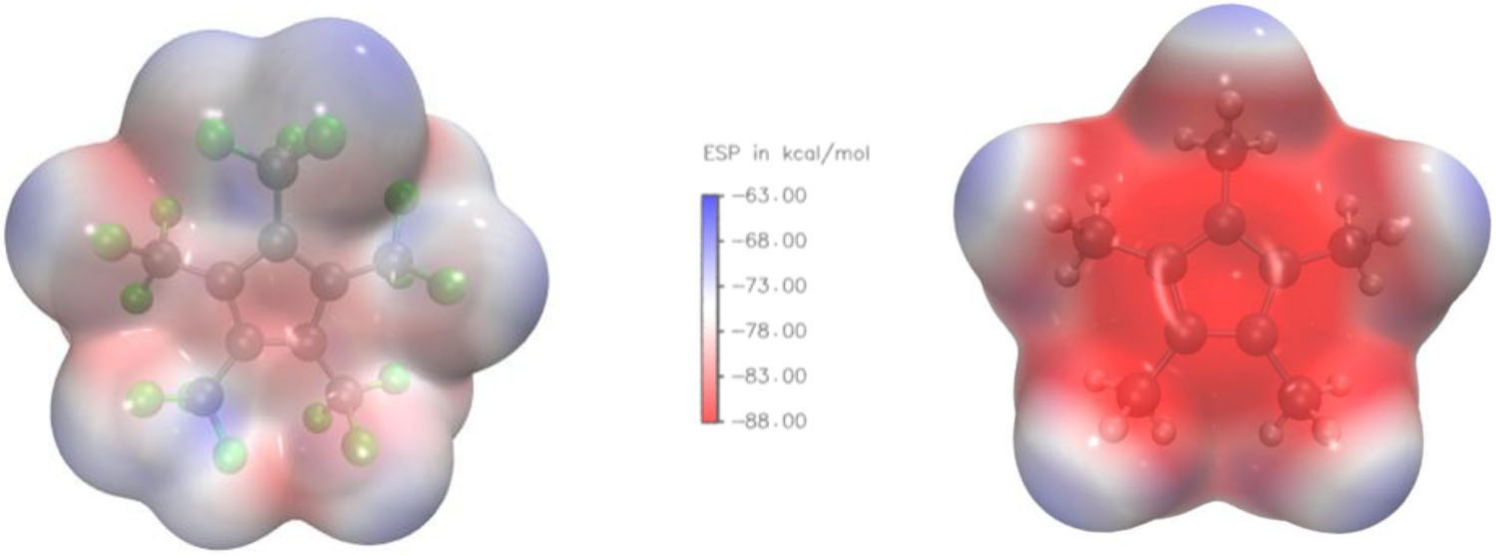
Electrostatic potential of [C_5_(CF_3_)_5_]^−^ (left) and [C_5_(CH_3_)_5_]^−^ (right) with an isodensity surface of 0.001 e/bohr^3^.

The weak coordination ability is illustrated by the electrostatic potential (ESP) of [C_5_(CF_3_)_5_]^−^ that was calculated by density functional theory (DFT, PW6B95D3/Def2TZVP). The obtained isodensity surface is remarkably homogenous, due to a strong electron density shift from the aromatic C_5_ cycle to the electron withdrawing CF_3_‐groups (Figure 2, left). Consequently, the most electron‐rich region is located around the central Cp ring. In this context the absolute energetic minimum has an ESP of −82.8 kcal/mol which is almost identical to the value for BABTP of the Gessner group.^[^ ^47^ ^]^ The maxima (ESP of −66.4 kcal/mol) are located on the fluorine atoms of the CF_3_ groups. For the electron‐rich and pronounced carbanionic Cp* [C_5_(CH_3_)_5_]^−^, the most electron‐rich regions have ESPs of up to −135.2 kcal/mol. In contrast, the maxima are located on the hydrogen atoms with ESP values of up to −69.0 kcal/mol (Figure 2, right). Furthermore, the DFT calculated (PW6B95D3/Def2TZVP) gas phase proton affinity (GPPA) provides a rational value to estimate the remaining basicity of a given WCA. While the GPPA for [C_5_(CF_3_)_5_]^−^ was calculated to have a surprisingly low value of 280 kcal/mol, its electron‐rich counterpart [C_5_(CH_3_)_5_]^−^ yielded 352 kcal/mol. For similar WCCAs, such as BABTP and triflide, comparable GPPAs of 277 and 286 kcal/mol, respectively, were obtained.^[^ ^43^, ^47^ ^]^

**Scheme 2:**
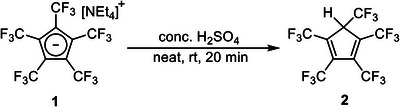
Formation of volatile and strongly acidic HC_5_(CF_3_)_5_ (**2**) from [NEt_4_][C_5_(CF_3_)_5_] (**1**) and concentrated H_2_SO_4_ for subsequent transfer reactions.

This is also emphasized by the very strong Brønsted acidity of the diene HC_5_(CF_3_)_5_. The conjugated acid of the perfluorinated Cp* has an extraordinary p*K*_a_ of −2.2 and is almost as acidic as concentrated H_2_SO_4_.^[^ ^56^, ^57^ ^]^ The combination of strong reactivity and thermal stability allows for its use as a powerful transfer reagent. Reaction of the air‐ and water‐stable [NEt_4_][C_5_(CF_3_)_5_] (**1**) with concentrated sulfuric acid yields HC_5_(CF_3_)_5_ (**2**) in almost quantitative yield (Scheme 2). The colorless and highly volatile diene is easily removed from the reaction mixture under vacuum and can be condensed onto any reactive precursor.

**Scheme 3:**
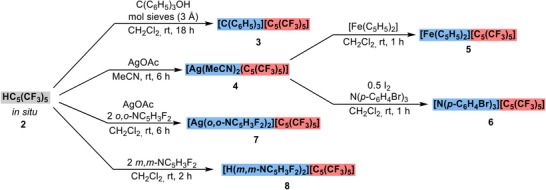
Synthesis of transfer reagents of the perfluorinated Cp* [C_5_(CF_3_)_5_]^−^ (red) with reactive cations (blue): [(C_6_H_5_)_3_C]^+^ (**3**), [Ag(MeCN)_2_]^+^ (**4**), [Fe(C_5_H_5_)_2_]^+^ (**5**), [N(*p*‐C_6_H_4_Br)_3_]^+^ (**6**), [Ag(*o,o*‐NC_5_H_3_F_2_)_2_]^+^ (**7**), [H(*m,m*‐NC_5_H_3_F_2_)_2_]^+^ (**8**).

**Figure 3:**
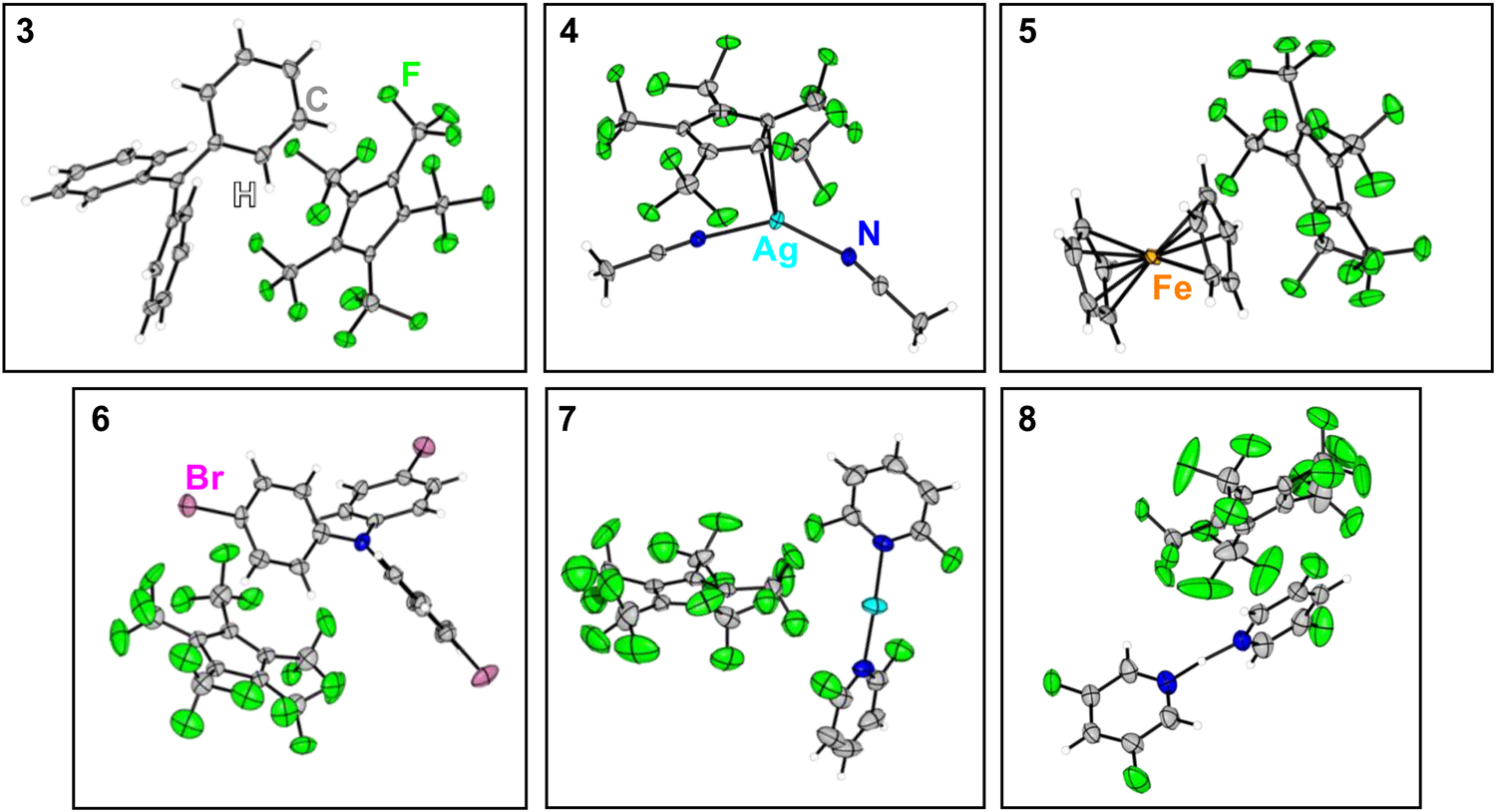
Molecular structures in the solid state of transfer reagents of the perfluorinated Cp* [C_5_(CF_3_)_5_]^−^ with reactive cations: [(C_6_H_5_)_3_C]^+^ (**3**), [Ag(MeCN)_2_]^+^ (**4**), [Fe(C_5_H_5_)_2_]^+^ (**5**), [N(*p*‐C_6_H_4_Br)_3_]^+^ (**6**), [Ag(*o,o*‐NC_5_H_3_F_2_)_2_]^+^ (**7**), [H(*m,m*‐NC_5_H_3_F_2_)_2_]^+^ (**8**). Disorder and solvent molecules are omitted for clarity. Ellipsoids are depicted with a 50% probability level. Color code: white‐hydrogen, grey‐carbon, green‐fluorine, light blue‐silver, deep blue‐nitrogen, orange‐iron, purple‐bromine.

For example, HC_5_(CF_3_)_5_ (**2)** was reacted with (C_6_H_5_)_3_COH to protonate and displace the hydroxyl group and generate the corresponding tritylium cation (Scheme 3). Removal of the formed water by the presence of 3 Å molecular sieves facilitates the complete conversion to the deep red [(C_6_H_5_)_3_C][C_5_(CF_3_)_5_] (**3**). Such tritylium cations are synthetically valuable transfer reagents for hydride and small anion (e.g., F^−^, CH_3_^−^, OH^−^) abstraction.^[^ ^58^, ^59^, ^60^, ^61^ ^] 19^F NMR spectroscopy showed a singlet at −50.7 ppm, indicating no C‐C bonding interaction between the perfluorinated Cp* and [(C_6_H_5_)_3_C]^+^ that would lead to distinct splitting patterns. This is further supported by the ^13^C{^1^H} NMR resonance shift of the tertiary carbenium at 211.1 ppm, since covalent bonding tends to yield values below 100 ppm for tritylium cations.^[^ ^62^ ^]^ Single crystals suitable for X‐ray diffraction (XRD) were obtained by slowly cooling a solution of *n*‐pentane/CH_2_Cl_2_ to −70 C. [(C_6_H_5_)_3_C][C_5_(CF_3_)_5_] (**3**) crystallized in the monoclinic *P*2_1_/*n* space group, demonstrating the ionic nature of the compound (Figure 3). Notably, this salt is only accessible, due to the low basicity of [C_5_(CF_3_)_5_]^−^, as normal Cp anions are known to form strong covalent C─C bonds with carbenium ions.^[^ ^63^, ^64^ ^]^

Other valuable transfer reagents within both the WCA and Cp chemistry are their respective alkali metal and especially lithium salts. Attempts to generate unstabilized [M][C_5_(CF_3_)_5_] (M = Li, Na, K) have been unsuccessful so far, due to the high fluoride ion affinity (FIA) and subsequent alkali fluoride elimination. However, an alternative was achieved by protonating AgOAc with HC_5_(CF_3_)_5_ in MeCN (Scheme 3). The release of HOAc gave stable [Ag(MeCN)_2_(C_5_(CF_3_)_5_)] (**4**) as a colorless solid, that is apparently stabilized by solvation, resulting in a decreased FIA of Ag(I). The existence as a solvate is indicated by the ^1^H NMR spectrum, which shows a shift of 2.24 ppm for the coordinated MeCN (compared to free MeCN at 1.97 ppm).^[^ ^65^ ^]^ The ^19^F NMR spectrum again showed a sharp singlet at −52.0 ppm. For transition metals, however, the questionable coordination/ionicity is difficult to resolve in terms of chemical shift and multiplicities, due to rapid metallotropic rearrangements.^[^ ^52^ ^]^ Single crystals suitable for XRD analysis were obtained by slowly cooling solutions of *n*‐pentane/CH_2_Cl_2_ to −70°C. [Ag(MeCN)_2_(C_5_(CF_3_)_5_)] (**4**) crystallized in the monoclinic *P*2_1_/*n* space group (Figure 3). The molecular structure in the solid state revealed the presence of two MeCN ligands and proved a weak interaction between [C_5_(CF_3_)_5_]^−^ and Ag(I). The Cp‐Ag bonding features a *η* ^2^‐hapticity with rather elongated Ag‐C distances of 2.701(3) and 2.723(4) Å, respectively. This result was surprising, since the perfluorinated Cp* is usually completely non‐competitive in coordinating solvents (e.g., MeCN, THF, Et_2_O). However, its unexpected affinity for coinage metals was subsequently investigated and demonstrated by our synthesis of stable [M(P*^t^* Bu_3_)(C_5_(CF_3_)_5_)] (M = Cu, Ag, Au) complexes.^[^ ^52^ ^]^ In general, the coexistence of cyclopentadienyls with silver is an extremely rare exception, and including our previous work, only two examples are known. This is mainly explained due to the strong oxidative character of Ag(I) and its associated reactivity as a one‐electron oxidizing agent.^[^ ^52^, ^66^ ^]^

While the presence of MeCN may compete with [C_5_(CF_3_)_5_]^−^ in subsequent reactions involving transition metals, we envisaged its synthetic value in the generation of other transfer reagents. For example, oxidation of ferrocene with [Ag(MeCN)_2_(C_5_(CF_3_)_5_)] (**4**) yielded deep green [Fe(C_5_H_5_)_2_][C_5_(CF_3_)_5_] (**5**), which is a mild oxidant with a potential of *E*_1/2_ = +0.40 V (versus SHE) according to the literature (Scheme 3).^[^ ^67^ ^]^ Single crystals suitable for XRD analysis were obtained by slowly cooling solutions of *n*‐pentane/CH_2_Cl_2_ to −70°C. [Fe(C_5_H_5_)_2_][C_5_(CF_3_)_5_] (**5**) is an ionic compound and crystallizes in the triclinic space group *P* $\bar{1}$ (Figure 3). The purity was confirmed by elemental analysis.

The oxidation power of Ag(I) salts can be further enhanced by using the synergistic Ag(I)/0.5 I_2_ system.^[^ ^68^, ^69^ ^]^ With the addition of elemental iodine, [Ag(MeCN)_2_(C_5_(CF_3_)_5_)] (**4**) was capable to oxidize [N(*p*‐C_6_H_4_Br)_3_] to the ammoniumyl radical cation [N(*p*‐C_6_H_4_Br)_3_][C_5_(CF_3_)_5_] (**6**) (Scheme 3). [N(*p*‐C_6_H_4_Br)_3_]^+^, known as “magic blue” is a potent and innocent one‐electron oxidizer with an literature‐known redox potential of *E*_1/2_ = +1.10 V (versus SHE).^[^ ^70^ ^]^ [N(*p*‐C_6_H_4_Br)_3_][C_5_(CF_3_)_5_] (**6**) represents an unprecedented example of an ammoniumyl Cp salt and also includes the most strongly oxidizing cation coexisting with a Cp anion. This can be explained by the extremely high ionization energy of [C_5_(CF_3_)_5_]^−^ compared to [C_5_H_5_]^−^ with 4.84 and 1.73 eV, respectively.^[^ ^52^, ^71^ ^]^ Single crystals suitable for XRD analysis were obtained by slowly cooling solutions of SO_2_ClF (due to micro‐crystallinity in organic solvents). [N(*p*‐C_6_H_4_Br)_3_][C_5_(CF_3_)_5_] (**6**) ∙ SO_2_ClF crystallized in the orthorhombic space group *Pna*2_1_ and showed separated cations and anions (Figure 3). Again, the purity was confirmed by elemental analysis.

In addition to the demonstrated utility of [Ag(MeCN)_2_(C_5_(CF_3_)_5_)] (**4**) for the preparation of oxidizing transfer reagents, the synthesis of a purely ionic Ag(I) salt with [C_5_(CF_3_)_5_]^−^, involving no competing co‐ligands was still pursued. By reacting HC_5_(CF_3_)_5_ (**2**) with AgOAc in CH_2_Cl_2_ (instead of MeCN), rapid decomposition was observed. However, when 2,6‐difluoropyridine (*o,o*‐NC_5_H_3_F_2_) was added to the reaction, stable [Ag(*o,o*‐NC_5_H_3_F_2_)_2_][C_5_(CF_3_)_5_] (**7**) was formed (Scheme 3). The ^19^F NMR spectrum showed a singlet at −52.1 ppm and a shifted doublet (compared to free *o,o*‐NC_5_H_3_F_2_) at −65.1 ppm for [C_5_(CF_3_)_5_]^−^ and the four *ortho*‐fluorine atoms, respectively. Single crystals suitable for XRD analysis were obtained by slowly cooling solutions of *n*‐pentane/CH_2_Cl_2_ to −70°C. [Ag(*o,o*‐NC_5_H_3_F_2_)_2_][C_5_(CF_3_)_5_] (**7**) crystallized in the triclinic *P* $\bar{1}$ space group. Interestingly, the [C_5_(CF_3_)_5_]^−^ moiety exists as a counteranion and is not coordinated to Ag(I), in contrast to the *η* ^2^‐coordinated [Ag(MeCN)_2_(C_5_(CF_3_)_5_)] (**4**) (Figure 3). Since MeCN is generally a stronger donor ligand than *o,o*‐NC_5_H_3_F_2_ and the perfluorinated Cp* has significant δ‐acceptor properties,^[^ ^51^, ^53^ ^]^ this difference can be explained by a synergistic push‐pull system for [Ag(MeCN)_2_(C_5_(CF_3_)_5_)] (**4**). Although not solvate‐free, *o,o*‐NC_5_H_3_F_2_ sufficiently stabilizes Ag(I), while exhibiting greatly reduced nucleophilicity compared to MeCN. This makes [Ag(*o,o*‐NC_5_H_3_F_2_)_2_][C_5_(CF_3_)_5_] (**7**) a synthetically valuable transfer reagent for organometallic chemistry for both oxidation and halide abstraction (Figure 3).

Since the diene HC_5_(CF_3_)_5_ (**2**) is a highly volatile liquid, stochiometric use and handling may be difficult. Therefore, we sought to generate a convenient Brønsted‐acidic equivalent. When HC_5_(CF_3_)_5_ (**2**) is reacted with an excess of 3,5‐difluoropyridine (*m,m*‐NC_5_H_3_F_2_) in CH_2_Cl_2_ the corresponding pyridinium salt [H(*m,m*‐NC_5_H_3_F_2_)_2_][C_5_(CF_3_)_5_] (**8**) is obtained in quantitative yield (Scheme 3). Here, the use of *o,o*‐NC_5_H_3_F_2_ and higher degrees of fluorination lead to unstable products. However, the *meta*‐fluorination of the pyridine is crucial to maintain a high Brønsted acidity, with a predicted p*K*_a_ = 0.40.^[^ ^72^ ^]^ The ^19^F NMR spectrum showed a singlet at −51.0 ppm and the doublet of the four *meta*‐fluorines at −118.6 ppm. In addition to the expected pyridinium resonances, the acidic proton singlet is resolved at 13.20 ppm in the ^1^H NMR spectrum. Single crystals suitable for XRD analysis were obtained by slowly cooling solutions of MeCN to −35°C. [H(*m*,*m*‐NC_5_H_3_F_2_)_2_][C_5_(CF_3_)_5_] (**8**) crystallizes in the monoclinic *P*2_1_/*c* space group and reveals the ionic nature of the compound (Figure 3). Due to the short N–N distance of 2.610(6) Å a structure with two short N─H bonds can be deduced, although the exact localization of the proton is associated with significant inaccuracies due to the only moderate quality of the structure. [H(*m*,*m*‐NC_5_H_3_F_2_)_2_][C_5_(CF_3_)_5_] (**8**) is a colorless solid that is storable and easy to handle. As a strong Brønsted acid equivalent, it may be used in similar protonation reactions as demonstrated with HC_5_(CF_3_)_5_ (**2**).

## 3 Conclusion

In conclusion, we report the synthesis and full characterization of unprecedented transfer reagents consisting of the perfluorinated Cp* [C_5_(CF_3_)_5_]^−^ and reactive cations generated from its extremely acidic diene HC_5_(CF_3_)_5_ (**2**). These include hydride‐abstracting [(C_6_H_5_)_3_C]^+^ (**3**), valuable Ag(I) salts (**4** and **7**), oxidizing [Fe(C_5_H_5_)_2_]^+^ (**5**) or [N(*p*‐C_6_H_4_Br)_3_]^+^ (**6**) and the convenient Brønsted acidic equivalent [H(*m*,*m*‐NC_5_H_3_F_2_)_2_]^+^ (**8**). The existence of these unseen ion pairs demonstrates the extremely low nucleophilicity and high oxidative resistance of the perfluorinated Cp*, allowing its introduction into the group of WCAs (and WCCAs). This is in strong contrast to the pronounced carbanionic character of common Cp anions, making [C_5_(CF_3_)_5_]^−^ an exception for both Cp and WCA chemistry. This is also illustrated by the calculated ESPs and the extraordinarily low GPPA, which is 72 kcal/mol lower than for the electron‐rich [C_5_(CH_3_)_5_]^−^. Since metal complexes of the perfluorinated Cp* ligand have been shown in the past to form unique ligand‐WCA switches but are difficult to access, the reactive transfer reagents described should increase the synthetic accessibility of these unusual metal complexes in the future.

## Supporting Information

The authors have cited additional references within the Supporting Information.^[^ ^73^, ^74^, ^75^, ^76^, ^77^, ^78^, ^79^, ^80^, ^81^, ^82^, ^83^, ^84^, ^85^, ^86^, ^87^, ^88^, ^89^, ^90^, ^91^, ^92^, ^93^, ^94^, ^95^, ^96^, ^97^, ^98^ ^]^

Deposition Number https://www.ccdc.cam.ac.uk/services/structures?id=https://doi.org/10.1002/chem.202500743 2,426,566 (for **3**), 2,426,567 (for **4**), 2,426,568 (for **5**), 2,426,569 (for **6**), 2,426,570 (for **7**) and 2,426,571 (for **8**) contain the supplementary crystallographic data for this paper. These data are provided free of charge by the joint Cambridge Crystallographic Data Centre and Fachinformationszentrum Karlsruhe http://www.ccdc.cam.ac.uk/structures Access Structures service.

## Conflict of Interests

The authors declare no conflict of interest.

## Supporting information



Supporting Information

## Data Availability

The data that support the findings of this study are available in the supplementary material of this article.

## References

[1] S. H. Strauss , Chem. Rev. 1993, 93, 927.

[2] I. Krossing , I. Raabe , Angew. Chem. Int. Ed. 2004, 43, 2066. 10.1002/anie.200300620 15083452

[3] T. A. Engesser , M. R. Lichtenthaler , M. Schleep , I. Krossing , Chem. Soc. Rev. 2016, 45, 789. 26612538 10.1039/c5cs00672d PMC4758321

[4] I. Riddlestone , A. Kraft , J. Schaefer , I. Krossing , Angew. Chem. Int. Ed. 2018, 57, 13982. 10.1002/anie.201710782 29266644

[5] M. R. Rosenthal , J. Chem. Educ. 1973, 50, 331.

[6] B. J. Hathaway , A. E. Underhill , J. Chem. Soc. 1961, 3091.

[7] N. M. N. Gowda , S. B. Naikar , G. K. N. Reddy , Adv. Inorg. Chem. 1984, 28, 255.

[8] W. Cockman , B. F. Hoskins , M. J. McCormick , T. A. Odonnell , Inorg. Chem. 1988, 27, 2742.

[9] W. H. Hersh , J. Am. Chem. Soc. 1985, 107, 4599.

[10] K. Shelly , T. Bartczak , W. R. Scheidt , C. A. Reed , Inorg. Chem. 1985, 24, 4325.

[11] T. Klapötke , U. Thewalt , J. Organomet. Chem. 1988, 356, 173.

[12] B. Buss , W. Clegg , G. Hartmann , P. G. Jones , R. Mews , M. Noltemeyer , G. M. Sheldrick , J. Chem. Soc. Dalton Trans. 1981, 61.

[13] K. Shelly , C. A. Reed , Y. J. Lee , W. R. Scheidt , J. Am. Chem. Soc. 1986, 108, 3117.

[14] H. P. A. Mercier , J. C. P. Saunders , G. T. Schrobilgen , J. Am. Chem. Soc. 1994, 116, 2921.

[15] S. Körbe , P. J. Schreiber , J. Michl , Chem. Rev. 2006, 106, 5208. 17165686 10.1021/cr050548u

[16] A. G. Massey , A. J. Park , J. Organomet. Chem. 1964, 2, 245.

[17] H. Nishida , N. Takada , M. Yoshimura , T. Sonoda , H. Kobayashi , Bull. Chem. Soc. Jpn. 1984, 57, 2600.

[18] D. M. van Seggen , P. K. Hurlburt , M. D. Noirot , O. P. Anderson , S. H. Strauss , Inorg. Chem. 1992, 31, 1423.

[19] A. Wiesner , T. W. Gries , S. Steinhauer , H. Beckers , S. Riedel , Angew. Chem. Int. Ed. 2017, 56, 8263. 10.1002/anie.201702807 28558157

[20] S. Kotsyuda , A. N. Toraman , P. Voßnacker , M. A. Ellwanger , S. Steinhauer , C. Müller , S. Riedel , Chem. Eur. J. 2022, 29, e202202749. 36268910 10.1002/chem.202202749 PMC10107151

[21] K. F. Hoffmann , A. Wiesner , N. Subat , S. Steinhauer , S. Riedel , Z. Anorg. Allg. Chem. 2018, 644, 1344.

[22] A. Wiesner , L. Fischer , S. Steinhauer , H. Beckers , S. Riedel , Chem. Eur. J. 2019, 25, 10441. 31090983 10.1002/chem.201901651

[23] D. M. van Seggen , P. K. Hurlburt , O. P. Anderson , S. H. Strauss , Inorg. Chem. 1995, 34, 3453.

[24] T. S. Cameron , I. Krossing , J. Passmore , Inorg. Chem. 2001, 40, 4488. 11487361 10.1021/ic010217+

[25] I. Krossing , Chem. Eur. J. 2001, 7, 490. 11271536 10.1002/1521-3765(20010119)7:2<490::aid-chem490>3.0.co;2-i

[26] C. Armbruster , M. Sellin , M. Seiler , T. Würz , F. Oesten , M. Schmucker , T. Sterbak , J. Fischer , V. Radtke , J. Hunger , et al., Nat. Commun. 2024, 15, 6721. 39112470 10.1038/s41467-024-50669-3 PMC11306567

[27] J. Willrett , M. Schmitt , V. Zhuravlev , M. Sellin , P. J. Malinowski , I. Krossing , Angew. Chem. Int. Ed. 2024, 63, e202405330. 10.1002/anie.202405330 38859637

[28] J. M. Rall , L. Nork , T. A. Engesser , M. Mayländer , S. Weber , S. Richert , I. Krossing , Chem. Eur. J. 2024, 30, e202400105. 38299788 10.1002/chem.202400105

[29] M. Sellin , I. Krossing , Acc. Chem. Res. 2023, 56, 2776. 37668537 10.1021/acs.accounts.3c00366

[30] A. Martens , P. Weis , M. Krummer , M. Kreuzer , A. Meierhöfer , S. C. Meier , J. Bohnenberger , H. Scherer , I. Riddlestone , I. Krossing , Chem. Sci. 2018, 9, 7058. 30310626 10.1039/c8sc02591f PMC6137444

[31] M. Sellin , J. Willrett , D. Röhner , T. Heizmann , J. Fischer , M. Seiler , C. Holzmann , T. A. Engesser , V. Radtke , I. Krossing , Angew. Chem. Int. Ed. 2024, 63, e202406742. 10.1002/anie.202406742 38842522

[32] S. Bulut , P. Klose , I. Krossing , Dalton Trans. 2011, 40, 8114. 21769367 10.1039/c1dt10722d

[33] L. Zapf , U. Radius , M. Finze , Dalton Trans. 2023, 52, 9553. 37203363 10.1039/d3dt01249b

[34] L. Zapf , M. Finze , Angew. Chem. Int. Ed. 2024, 63, e202401681. 10.1002/anie.202401681 38530744

[35] E. Bernhardt , M. Finze , H. Willner , C. W. Lehmann , F. Aubke , Angew. Chem. Int. Ed. 2003, 42, 2077. 10.1002/anie.200250780 12746828

[36] M. Finze , E. Bernhardt , H. Willner , C. W. Lehmann , Angew. Chem. Int. Ed. 2004, 43, 4160. 10.1002/anie.200454034 15307076

[37] N. Tiessen , B. Neumann , H. G. Stammler , B. Hoge , Chem. Eur. J. 2020, 26, 13611. 32196783 10.1002/chem.202000668 PMC7693355

[38] S. Porath , M. Keßler , B. Neumann , H. G. Stammler , B. Hoge , Chem. Eur. J. 2023, 29, e20220327. 10.1002/chem.202203278 36610041

[39] K. Tölke , B. Neumann , H. G. Stammler , B. Hoge , Chem. Eur. J. 2024, 30, e202403226. 39298331 10.1002/chem.202403226

[40] T. H. Meaghan , A. Bruening , M. Espinosa , T. Agapie , Angew. Chem. Int. Ed. 2025, 64, e202417136. 10.1002/anie.202417136 39401945

[41] C. Schulz , J. Daniels , T. Bredow , J. Beck , Angew. Chem. Int. Ed. 2016, 55, 1173. 10.1002/anie.201507644 26632775

[42] K. Searles , K. Keijzer , C. H. Chen , M. H. Baik , D. J. Mindiola , Chem. Commun. 2014, 50, 6267. 10.1039/c4cc01404a 24788367

[43] L. Turowsky , K. Seppelt , Inorg. Chem. 1988, 27, 2135.

[44] K. Ishihara , A. Hasegawa , H. Yamamoto , Angew. Chem. Int. Ed. 2001, 40, 4077. 10.1002/1521-3773(20011105)40:21<4077::AID-ANIE4077>3.0.CO;2-1 29712252

[45] A. Hasegawa , T. Ishikawa , K. Ishihara , H. Yamamoto , Bull. Chem. Soc. Jpn. 2005, 78, 1401.

[46] D. Höfler , M. van Gemmeren , P. Wedemann , K. Kaupmees , I. Leito , M. Leutzsch , J. B. Lingnau , B. List , Angew. Chem. Int. Ed. 2017, 56, 1411. 10.1002/anie.201609923 28004482

[47] L. Kelling , J. Eßer , D. Knyszek , V. H. Gessner , Angew. Chem. Int. Ed. 2024, 63, e202405936. 10.1002/anie.202405936 38877830

[48] L. Kelling , V. H. Gessner , Polyhedron 2025, 265, 117283.

[49] K. Hazin , B. O. Patrick , D. P. Gates , Inorg. Chem. 2019, 58, 188. 30525516 10.1021/acs.inorgchem.8b02174

[50] B. Peng , J. Ma , J. Guo , Y. Gong , R. Wang , Y. Zhang , J. Zeng , W. W. Chen , K. Ding , B. Zhao , J. Am. Chem. Soc. 2022, 144, 2853. 35143204 10.1021/jacs.1c12723

[51] R. Sievers , M. Sellin , S. M. Rupf , J. Parche , M. Malischewski , Angew. Chem. Int. Ed. 2022, 61, e202211147. 10.1002/anie.202211147 PMC9826324 35984742

[52] R. Sievers , M. Reimann , N. G. Kub , S. M. Rupf , M. Kaupp , M. Malischewski , Chem. Sci. 2024, 15, 2990. 38404370 10.1039/d3sc06299f PMC10882543

[53] R. Sievers , J. Parche , N. G. Kub , M. Malischewski , Synlett 2023, 34, 1079.

[54] J. Parche , S. M. Rupf , R. Sievers , M. Malischewski , Dalton Trans. 2023, 52, 5496. 37006118 10.1039/d3dt00425b

[55] N. G. Kub , R. Sievers , J. Parche , M. Malischewski , Chem. Eur. J. 2024, 30, e202400427. 38380762 10.1002/chem.202400427

[56] E. D. Laganis , D. M. Lemal , J. Am. Chem. Soc. 1980, 102, 6633.

[57] G. Paprott , K. Seppelt , J. Am. Chem. Soc. 1984, 106, 4060.

[58] Y. Gong , F. F. Mulks , Synthesis 2025, 57, A‐I.

[59] M. Horn , H. Mayr , J. Phys. Org. Chem. 2012, 25, 979.

[60] R. R. Naredla , D. A. Klumpp , Chem. Rev. 2013, 113, 6905. 23819438 10.1021/cr4001385

[61] K. F. Hoffmann , D. Battke , P. Golz , S. M. Rupf , M. Malischewski , S. Riedel , Angew. Chem. Int. Ed. 2022, 61, e202203777. 10.1002/anie.202203777 PMC9401592 35416383

[62] A. Hinz , R. Labbow , F. Reiß , A. Schulz , K. Sievert , A. Villinger , Struct. Chem. 2015, 26, 1641.

[63] E. A. Bielinski , W. Dai , L. M. Guard , N. Hazari , M. K. Takase , Organometallics 2013, 32, 4025.

[64] J. D. White , T. Furuta , M. McCamish , Synth. Commun. 1973, 3, 425.

[65] G. R. Fulmer , A. J. M. Miller , N. H. Sherden , H. E. Gottlieb , A. Nudelman , B. M. Stoltz , J. E. Bercaw , K. I. Goldberg , Organometallics 2010, 29, 2176.

[66] H. G. Stammler , P. Jutzi , W. Wieland , B. Neumann , Acta Crystallogr., Sect. C:Cryst. Struct. Commun. 1998, 54, IUC9800064.

[67] A. Paul , R. Borrelli , H. Bouyanfif , S. Gottis , F. Sauvage , ACS Omega 2019, 4, 14780. 31552317 10.1021/acsomega.9b01341 PMC6751539

[68] P. J. Malinowski , D. Himmel , I. Krossing , Angew. Chem. Int. Ed. 2016, 55, 9259. 10.1002/anie.201603741 27404568

[69] V. Zhuravlev , P. J. Malinowski , Angew. Chem. Int. Ed. 2018, 130, 11871. 10.1002/anie.201806836 30020562

[70] X. Wu , A. P. Davis , P. C. Lambert , L. K. Steffen , O. Toy , A. J. Fry , Tetrahedron 2009, 65, 2408.

[71] P. K. Lo , K. C. Lau , J. Phys. Chem. 2014, 118, 2498. 10.1021/jp412323j 24621131

[72] K. Clarke , K. Rothwell , J. Chem. Soc. 1960, 1885.

[73] H. E. Gottlieb , V. Kotlyar , A. Nudelman , J. Org. Chem. 1997, 62, 7512. 11671879 10.1021/jo971176v

[74] R. K. Harris , E. D. Becker , S. M. Cabral de Menezes , R. Goodfellow , P. Granger , Pure Appl. Chem. 2001, 73, 1795.

[75] M. R. Willcott , J. Am. Chem. Soc. 2009, 131, 13180.

[76] O. V. Dolomanov , L. J. Bourhis , R. J. Gildea , J. A. K. Howard , H. Puschmann , J. Appl. Cryst. 2009, 42, 339. 10.1107/S0021889811041161 PMC3236671 22199401

[77] G. M. Sheldrick , Acta Crystallogr., Sect. A 2015, A71, 3.

[78] G. M. Sheldrick , SHELXL Version 2014/7, Program for Crystal Structure Solution and Refinement, Göttingen, Germany, 2014.

[79] G. M. Sheldrick , Acta Crystallogr., Sect. A 2008, A64, 112.

[80] K. Brandenburg , Diamond: Crystal and Molecular Structure Visualization, http://www.crystalimpact.com/diamond (accessed:)

[81] Persistence of Vision Pty. Ltd . Persistence of Vision Raytracer. Ltd., Persistence of Vision Pty. 2004. http://www.povray.org

[82] P. Hohenberg , W. Kohn , Phys. Rev. 1964, 136, B864.

[83] W. Kohn , L. J. Sham , Phys. Rev. 1965, 140, A1133.

[84] M. J. Frisch , G. W. Trucks , H. B. Schlegel , G. E. Scuseria , M. A. Robb , J. R. Cheeseman , G. Scalmani , V. Barone , G. A. Petersson , H. Nakatsuji , X. Li , M. Caricato , A. V. Marenich , J. Bloino , B. G. Janesko , R. Gomperts , B. Mennucci , H. P. Hratchian , J. V. Ortiz , A. F. Izmaylov , J. L. Sonnenberg , D. Williams‐Young , F. Ding , F. Lipparini , F. Egidi , J. Goings , B. Peng , A. Petrone , T. Henderson , D. Ranasinghe , et al. Gaussian 16, Revision C.02. Gaussian, Inc., Wallingford CT 2019.

[85] Y. Zhao , D. G. Truhlar , J. Phys. Chem. 2005, 109, 5656. 10.1021/jp050536c 16833898

[86] F. Weigend , R. Ahlrichs , Phys. Chem. Chem. Phys. 2005, 7, 3297. 16240044 10.1039/b508541a

[87] T. Lu , F. Chen , J. Comput. Chem. 2012, 33, 580. 22162017 10.1002/jcc.22885

[88] J. Zhang , T. Lu , Phys. Chem. Chem. Phys. 2021, 23, 20323. 34486612 10.1039/d1cp02805g

[89] W. Humphrey , A. Dalke , K. J. Schulten , J. Mol. Graphics 1996, 14, 33. 10.1016/0263-7855(96)00018-5 8744570

[90] E. P. Janulis , A. J. Arduengo , J. Am. Chem. Soc. 1983, 105, 3563.

[91] R. D. Chambers , W. K. Gray , J. F. S. Vaughan , S. R. Korn , M. Médebielle , A. S. Batsanov , C. W. Lehmann , J. A. K. Howard , Perkin Trans. 1997, 135.

[92] R. Sievers , M. Sellin , S. M. Rupf , M. Malischewski , Angew. Chem. Int. Ed. 2022, 61, e202211147. 10.1002/anie.202211147 PMC9826324 35984742

[93] Trace impurities: 2.98 and 1.21 ppm (1 mol% [NEt4][C5(CF3)5]), 0.08 ppm (H‐grease).

[94] [C5(CF3)5]− resonances are supressed and invisible in ^13^C NMR spectroscopy even at prolonged measurement times.

[95] Trace impurities: 2.08 (acetone from CD_2_Cl_2_).

[96] Trace impurities: 2.08 (acetone from CD_2_Cl_2_), 1.26 and 0.88 ppm (*n*‐pentane from CD_2_Cl_2_), 0.08 ppm (H‐grease).

[97] Trace impurities: 1.26 and 0.88 ppm (*n*‐pentane from CD_2_Cl_2_).

[98] The high *R*‐values originate from pronounced disordering of the CF_3_‐groups around the C‐C axis.

