# Supplementary material for: Coexistence of the Perfluorinated Cp* Anion With Oxidizing and Electrophilic Cations
Source: Chemistry. 2025 Apr 17;31(28):e202500743. doi: 10.1002/chem.202500743 (PMC12089903; doi:10.1002/chem.202500743)
Supplement: Supplementary file 1 — Supporting Information [file CHEM-31-e202500743-s001.pdf]

SUPPORTING INFORMATION

Coexistence of the Perfluorinated Cp* Anion with Oxidizing and Electrophilic Cations

Robin Sievers, Nico G. Kub, Tim-Niclas Streit, Susanne M. Rupf, Moritz Malischewski*

Abstract: The air- and water stable perfluorinated Cp* $[C_5(CF_3)_5]^-$ is presented as a candidate for the vastly underexplored group of weakly coordinating carbanions (WCCAs). Its extreme electron deficiency combined with the stabilization of the negative charge within an aromatic system results in a low basicity, yielding a very weak coordination ability. As Cp anions usually possess a strongly pronounced carbanionic character, the perfluorinated Cp* resembles an extraordinary exception for both the WCA and Cp chemistry. However, the coordination ability remains ambivalent due to the substitution lability of many of its complexes, allowing for the formation of unique ligand-WCA switches. Due to the low reactivity, there is a need for new transfer reagents containing the perfluorinated Cp* in combination with reactive cations. Thus, we report the synthesis and complete characterization of $[C_5(CF_3)_5]^-$ salts with hydride-accepting $[(C_6H_5)_3C]^+$, valuable Ag(I) reagents, oxidizing $[Fe(C_5H_5)_2]^+$ or $[N(p\text{-}C_6H_4Br)_3]^+$ and Brønsted acidic $[H(m,m\text{-}NC_5H_3F_2)_2]^+$. Notably, these unprecedented ion pairs are exclusively accessible and stabilized by the low coordination ability and pronounced oxidative resistance of the perfluorinated Cp*.

Contents

General Information 1
Synthetic Procedures 3
NMR Spectra 8
IR Spectra 16
Crystallographic Data 19
Density functional theory 28
References 32

General Information

Reactions and workups sensitive to air were performed in previously heated glassware under an atmosphere of argon using standard Schlenk techniques and an oil pump vacuum of 10^{-3} mbar. Room temperature (rt) refers to 25 °C. The addition of liquid reagents and solvents was done by using threefold argon-flushed disposable syringes and septa, while solids were added in argon stream. Low temperature reactions were performed in a cooled ethanol-bath. Glassware was cleaned by storing in a potassium hydroxide bath for several days, rinsed with diluted hydrochloric acid and doubly deionized water and dried at 150 °C.

Pressure reactions

The synthesis of $[NEt_4][C_5(CF_3)_5]$ involves high temperatures and highly volatile substances in a closed system. Hence, it must be assumed, that high pressures arise upon heating and advanced caution is required. Therefore, it is advisable to perform the reaction in a separate and properly closed fumehood. The thick-walled glass reaction vessel should not be opened and if possible, not even touched until the reaction has finished and reached rt.

Solvents, reagents and materials

Anhydrous MeCN, CH_2Cl_2 and *n*-pentane were obtained from the solvent system FMBRAUN MB SPS-800 and stored over activated 3 Å mol sieves. Deuterated solvents CD_2Cl_2 and CD_3CN were used as purchased and stored over activated 3 Å mol sieves. Sulfolane was heated at 60 °C for at least 24 h over activated 3 Å molecular sieves and additionally 1 h in high vacuum prior to use. 18-crown-6 was heated at 80 °C for 2 h in high vacuum prior to use. All other solvents and commercially available reagents were used without further purification.

Nuclear magnetic resonance (NMR) spectroscopy

NMR spectroscopy was measured on a JEOL ECX 400 (400 MHz) or a Varian INOVA 600 (600 MHz) in the reported deuterated solvents CD_2Cl_2 and CD_3CN. All given chemical shifts in ^{1}H-NMR spectra are calibrated on the resonance signals of $CDHCl_2$ contained in CD_2Cl_2 (δ = 5.32 ppm) and CD_2HCN (δ = 1.94 ppm) contained in CD_3CN. The ^{13}C-NMR spectra are calibrated on the respective resonance signals of CD_2Cl_2 (δ = 53.84 ppm) and CD_3CN (δ = 1.32 and 118.26 ppm).[65,73] The ^{19}F- and ^{31}P-NMR spectra are device-internally calibrated relative to the resonance signal of $CFCl_3$ and H_3PO_4 according to the unified chemical shift scale.[74] The given multiplicities are phenomenological, thus the actual appearance of the signals is stated and not the theoretically expected one. The following abbreviations were used and analogously combined to designate multiplicities: s (singlet), d (doublet), t (triplet), q (quartet), m (multiplet), m_c (centrosymmetric multiplet). For centrosymmetric multiplets the center and for non-symmetric multiplets the interval is stated. Evaluation of spectra was performed with Mestrelab Research MNova 7.[75]

Infrared (IR) spectroscopy

IR spectroscopy was measured on a FT (Fourier transformation) Nicolet iS10 IR-spectrometer. The sample was directly measured by ATR (attenuated total reflection) technique. Characteristic absorptions are given in wavenumbers $\tilde{\nu}$ [cm^{-1}] and intensities are stated as vs (very strong), s (strong), m (medium) and w (weak).

High resolution mass spectrometry (HRMS) and elemental analysis (EA)

HRMS was recorded using an AGILENT 6210 spectrometer by electrospray ionization (ESI) at the department of mass spectrometry at the Freie Universität Berlin. A detailed listing of fragmentation is dispensed, instead the molecular ion peak or a characteristic fragment peak is stated. EA was measured on a VARIO EL. Relative proportion of C, H and N are given in percent.

X-ray diffraction (XRD)

X-Ray data were collected on a BRUKER D8 Venture system. Data were collected at 100(2) K using graphite monochromated Mo K_{α} radiation (λ_{α} = 0.71073 Å). The strategy for the data collection was evaluated by using the Smart software. The data were collected by the standard "ψ-ω scan techniques" and were scaled and reduced using Saint+software. The structures were solved by using Olex2,[76] with the XT[77] structure solution program using Intrinsic Phasing and refined with the XL refinement package[78,79] using Least Squares minimization. Bond length and angles were measured with Diamond Crystal and Molecular Structure Visualization Version 4.6.2.[80] Drawings were generated with POV-Ray.[81] In all structures, hydrogen atoms were placed using constraints/a riding model. Only for H1 in compound **8** a found Q-peak was used instead. In several cases, significant disorder of the $[C_5(CF_3)_5]^-$ moiety is observed.

Density functional theory (DFT)

DFT calculations were performed with Gaussian 16,[82,83] Revision C.02,[84] using the PW6B95D3[85] functional and the Def2TZVP[86] basis set for structure optimizations, energy and frequency calculations without any solvent model. The gas phase proton affinities (GPPA) were calculated from the sum of electronic and thermal free energies: GPPA = $\Delta G_g^0(CH) - \Delta G_g^0(C^-) - \Delta G_g^0(H^+)$. From the optimized structures molecular surfaces in the form of grid patterns, as well as surface analysis/electrostatic potentials (ESP) were calculated using MultiWFN 3.8.[87,88] ESPs mapped on the molecular surfaces were depicted with the VMD 1.9.3.[89] An isovalue of 0.001 e/bohr^3 was used for the electron density with a spacing of grid points for the surface of 0.25 Bohr. The RWB colour code was used, showing maxima (electron-rich regions) in red and minima (electron-poor regions) in blue.

Synthetic Procedures

$[NEt_4][C_5(CF_3)_5]$ (1)

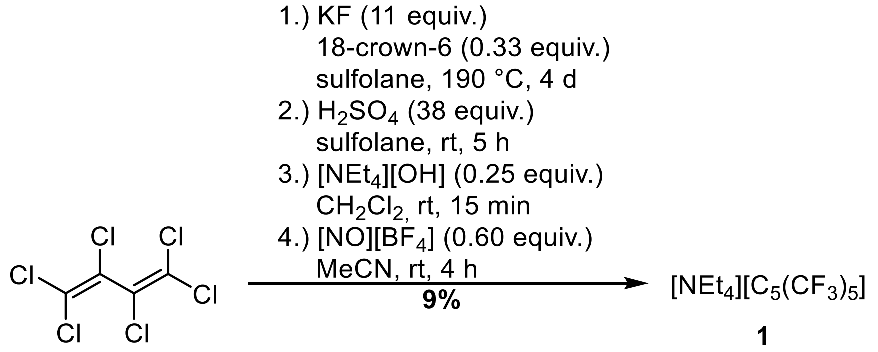

In a dried 1000 mL pressure flask anhydrous KF (60 g, 1.0 mol, 11 equiv.) was placed in anhydrous and degassed sulfolane (190 mL) under an atmosphere of argon. Anhydrous and degassed 18-crown-6 (8.7 g, 33 mmol, 0.33 equiv.) and hexachlorobuta-1,3-diene (15 mL, 96 mmol, 1.0 equiv.) were added at rt. The resulting reaction mixture was carefully shaken and cooled to –196 °C in high vacuum. The properly closed pressure flask was slowly warmed to 190 °C and stirred at this temperature for 3 d. Then the resulting black suspension was cooled to rt and the volatiles were removed in high vacuum. The remaining mixture was filtrated under an atmosphere of argon and the residue was extracted with anhydrous MeCN (3 × 40 mL). The filtrate was warmed to 40 °C and all MeCN was removed in high vacuum, while stirring. The resulting solution was put under high vacuum and H_2SO_4 (conc., 200 mL, 3.6 mol, 38 equiv.) was added dropwise at rt over a period of 3 h, while stirring and continuously collecting the volatiles in a cold trap of –196 °C. After complete addition, the mixture remained for additional 2 h in high vacuum. The cold trap was put under argon and slowly warmed to 0 °C, giving a pale yellow liquid. Then CH_2Cl_2 (20 mL) and a solution of $[NEt_4][OH]$ (35% in water, 10 mL, 24 mmol, 0.25 equiv.) were added and the reaction mixture was stirred for 15 min at rt, giving a deep red solution. The aqueous layer was separated and extracted with CH_2Cl_2 (4 × 20 mL). The combined organic layers were dried over $MgSO_4$, filtrated and the solvent was removed under reduced pressure. The remaining solid was suspended in Et_2O (~5 mL) and recrystallized twice from CH_2Cl_2 (~10 mL) by slowly cooling to –20 °C. The crystalline residue was decanted and washed with Et_2O (2 × 5 mL). The solvents were removed under reduced pressure to give a product mixture of 85 mol% $[NEt_4][C_5(CF_3)_5]$ (**1**, 1.7 g, 3.2 mmol) and 15 mol% $[NEt_4][C_5(CF_3)_4H]$ (0.30 g, 0.50 mmol) that was placed in a dried 50 mL Schlenk flask in anhydrous MeCN (20 mL). $[NO][BF_4]$ (0.26 g, 2.2 mmol, 0.60 equiv.) was added and the reaction mixture was stirred at room temperature for 4 h. Dest. H_2O (20 mL) was slowly added and stirred for 15 min before separating aqueous and organic phase. The aqueous phase was extracted with CH_2Cl_2 (3 × 20 mL). The combined organic phases were dried over $MgSO_4$, filtrated and the solvents removed under reduced pressure. The residue was dissolved in CH_2Cl_2 (1 mL) and slowly added to stirred *n*Bu_2O (200 mL). The colourless suspension was filtrated and the residue washed with *n*Bu_2O (2 × 10 mL) and n-pentane (2 × 10 mL). The solvent was removed under reduced pressure to give $[NEt_4][C_5(CF_3)_5]$ (**1**, 1.7 g, 3.2 mmol) as a colourless amorphous solid with a yield of 9%.

^{1}H NMR (400 MHz, CD_2Cl_2, rt) δ [ppm] = 2.98 (q, $^3J_{H,H}$ = 7.3 Hz, 8H), 1.21 (q, $^3J_{H,H}$ = 7.1 Hz, 12H). **^{19}F NMR** (377 MHz, CD_2Cl_2, rt) δ [ppm] = –50.6 (s, 15F). **^{13}C{^{1}H} NMR** (151 MHz, CD_2Cl_2, rt) δ [ppm] = 52.7 (m_c, 4C), 7.3 (s, 4C). **^{13}C{^{19}F} NMR** (151 MHz, CD_2Cl_2, rt) δ [ppm] = 125.4 (s, 5C), 110.2 (s, 5C). The analytical data are consistent with those reported in literature.[90–92]

[C(C$_6$H$_5$)$_3$][C$_5$(CF$_3$)$_5$] (3)

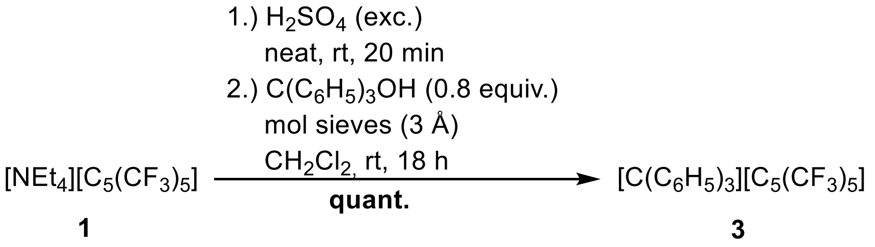

In a dried 10 mL Schlenk flask H_2SO_4 (conc., 2.0 mL, exc.) was placed under an argon atmosphere and cooled to −196 °C before $[NEt_4][C_5(CF_3)_5]$ (**1**, 0.10 g, 0.19 mmol, 1.0 equiv.) was added. Then the reaction mixture was warmed to rt under high vacuum and stirred at this temperature for 20 min. The volatiles were continuously trapped in a second dried 10 mL Schlenk flask cooled to −196 °C, containing $C(C_6H_5)_3OH$ (39 mg, 0.15 mmol, 0.8 equiv.) and activated 3 Å molecular sieves in anhydrous CH_2Cl_2 (2 mL). The second Schlenk flask was put under an atmosphere of argon, warmed to rt and stirred at this temperature for 18 h. The reaction mixture was filtered under an atmosphere of argon and the solvent was removed in high vacuum. The remaining solid was washed with anhydrous *n*-pentane (3 × 2 mL) and dried in high vacuum to give product $[C(C_6H_5)_3][C_5(CF_3)_5]$ (**3**, 97 mg, 0.15 mmol) as a red amorphous solid with a quantitative yield.

^{1}H NMR (400 MHz, CD_2Cl_2, rt) δ [ppm] = 8.24 (t, $^3J_{H,H}$ = 7.5 Hz, 3H), 7.85 (t, $^3J_{H,H}$ = 8.0 Hz, 6H), 7.63 (d, $^3J_{H,H}$ = 7.0 Hz, 6H).[93] **^{19}F NMR** (377 MHz, CD_2Cl_2, rt) δ [ppm] = –50.7 (s, 15F). **^{13}C{^{1}H} NMR** (101 MHz, CD_2Cl_2, rt) δ [ppm] = 211.1 (s, 1C), 143.9 (s, 3C), 143.0 (s, 6C), 140.2 (s, 3C), 130.9 (s, 6C).[94] **FT-IR** (ATR) ṽ [cm^{-1}] = 3065 (w), 1584 (s), 1484 (m), 1356 (s), 1296 (m), 1205 (vs), 1115 (vs), 996 (m), 978 (m), 948 (w), 915 (w), 890 (w), 843 (m), 806 (m), 768 (s), 700 (vs), 660 (w), 632 (s), 622 (s), 608 (s). **HRMS** (ESI-TOF, negative) m/z for $[C_{10}F_{15}]^-$ calculated: 404.9760; measured: 404.9873. **EA** for $[C_{29}H_{15}F_{15}]$ calculated: C: 53.72%, H: 2.33%; measured: C: 52.71%, H: 3.31%. A **molecular structure in solid state** was measured for this compound (table S1 and figure S23).

[Ag(MeCN)$_2$(C$_5$(CF$_3$)$_5$)] (4)

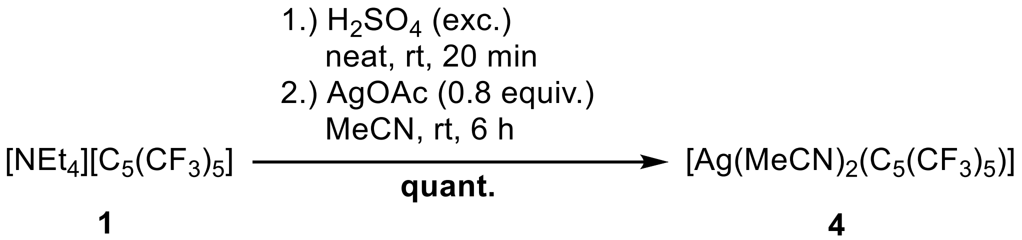

In a dried 10 mL Schlenk flask H_2SO_4 (conc., 2.0 mL, exc.) was placed under an argon atmosphere and cooled to −196 °C before $[NEt_4][C_5(CF_3)_5]$ (**1**, 0.10 g, 0.19 mmol, 1.0 equiv.) was added. Then the reaction mixture was warmed to rt under high vacuum and stirred at this temperature for 20 min. The volatiles were continuously trapped in a second dried 10 mL Schlenk flask cooled to −196 °C, containing AgOAc (25 mg, 0.15 mmol, 0.8 equiv.) in

anhydrous MeCN (2 mL). The second Schlenk flask was put under an atmosphere of argon, warmed to rt and stirred at this temperature for 6 h under the exclusion of light. The solvent was removed in high vacuum and the remaining solid was washed with anhydrous *n*-pentane (3 × 2 mL) and dried in high vacuum to give product $[Ag(MeCN)_2(C_5(CF_3)_5)]$ (**4**, 89 mg, 0.15 mmol) as a colorless amorphous solid with a quantitative yield.

^{1}H NMR (400 MHz, CD_2Cl_2, rt) δ [ppm] = 2.24 (s, 6H).[95] **^{19}F NMR** (377 MHz, CD_2Cl_2, rt) δ [ppm] = −52.0 (s, 15F). **^{13}C{^{19}F} NMR** (151 MHz, CD_2Cl_2, rt) δ [ppm] = 124.0 (m_c, 5C), 120.1 (q, $^2J_{C,H}$ = 10.2 Hz, 2C), 110.3 (s, 5C), 2.5 (q, $^1J_{C,H}$ = 138.4 Hz, 2C) . **FT-IR** (ATR) ṽ [cm^{-1}] = 2945 (w), 2314 (w), 2284 (w), 1657 (w), 1545 (w), 1493 (w), 1401 (w), 1367 (w), 1289 (w), 1202 (vs), 1112 (vs), 1035 (m), 938 (w), 802 (w), 769 (w), 726 (w), 632 (s), 603 (w). **HRMS** (ESI-TOF, negative) m/z for $[C_{10}F_{15}]^-$ calculated: 404.9760; measured: 404.9651. **EA** for $[C_{14}H_6F_{15}N_2Ag]$ calculated: C: 28.26%, H: 1.02%, N: 4.71%; measured: C: 27.77%, H: 1.41%, N: 5.96%. A **molecular structure in solid state** was measured for this compound (table S2 and figure S24).

$[Fe(C_5H_5)_2][C_5(CF_3)_5]$ (5)

$$[Ag(MeCN)_2(C_5(CF_3)_5)] \xrightarrow[CH_2Cl_2,\ rt,\ 1\ h]{[Fe(C_5H_5)_2]\ (1.1\ equiv.)} [Fe(C_5H_5)_2][C_5(CF_3)_5]$$

4   **quant.**   **5**

In a dried 10 mL Schlenk flask $[Ag(MeCN)_2(C_5(CF_3)_5)]$ (**4**, 0.10 g, 0.17 mmol, 1.0 equiv.) and $[Fe(C_5H_5)_2]$ (35 mg, 0.19 mmol, 1.1 equiv.) were dissolved in anhydrous CH_2Cl_2 (3 mL) and the reaction mixture was stirred at rt for 1 h. The reaction mixture was filtered under an atmosphere of argon and the solvent was removed in high vacuum. The remaining solid was washed with anhydrous *n*-pentane (3 × 2 mL) and dried in high vacuum to give product $[Fe(C_5H_5)_2][C_5(CF_3)_5]$ (**5**, 0.10 mg, 0.17 mmol) as a green amorphous solid with a quantitative yield.

FT-IR (ATR) ṽ [cm^{-1}] = 3128 (w), 1669 (w), 1493 (m), 1421 (w), 1340 (w), 1277 (w), 1198 (vs), 1090 (vs), 1005 (m), 950 (w), 881 (w), 852 (s), 800 (m), 742 (w), 695 (w), 659 (w), 632 (s). **HRMS** (ESI-TOF, negative) m/z for $[C_{10}F_{15}]^-$ calculated: 404.9760; measured: 404.9583. **EA** for $[C_{20}H_{10}F_{15}Fe]$ calculated: C: 40.64%, H: 1.71%; measured: C: 40.75%, H: 1.78%. A **molecular structure in solid state** was measured for this compound (table S3 and figure S25).

$[N(p\text{-}C_6H_4Br)_3][C_5(CF_3)_5]$ (6)

$$[Ag(MeCN)_2(C_5(CF_3)_5)] \xrightarrow[CH_2Cl_2,\ rt,\ 1\ h]{I_2\ (0.5\ equiv.)\quad [N(p\text{-}C_6H_4Br)]\ (1.1\ equiv.)} [N(p\text{-}C_6H_4Br)_3][C_5(CF_3)_5]$$

4   **quant.**   **6**

In a dried 10 mL Schlenk flask $[Ag(MeCN)_2(C_5(CF_3)_5)]$ (**4**, 100 mg, 0.17 mmol, 1.0 equiv.) and $[N(p\text{-}C_6H_4Br)_3]$ (92 mg, 0.19 mmol, 1.1 equiv.) were dissolved in anhydrous CH_2Cl_2 (3mL), I_2 (23 mg, 90 µmol, 0.5 equiv.) was added and the reaction mixture was stirred at rt for 1 h. The reaction mixture was filtered and the solvent was removed in high vacuum. The remaining solid was washed with anhydrous *n*-pentane (3 × 2 mL) and dried in high vacuum to give product $[N(p\text{-}C_6H_4Br)_3][C_5(CF_3)_5]$ (**6**, 0.15 g, 0.17 mmol) as a blue amorphous solid in quantitative yield.

FT-IR (ATR) $\tilde{\nu}$ [cm^{-1}] = 3098 (w), 1546 (s), 1494 (s), 1418 (m), 1205 (vs), 1109 (vs), 1062 (vs), 996 (s), 912 (m), 828 (vs), 768 (m), 632 (s), 569 (m). **HRMS** (ESI-TOF, negative) m/z for $[C_{10}F_{15}]^-$ calculated: 404.9760; measured: 404.9700. **EA** for $[C_{28}H_{12}F_{15}Br_3N]$ calculated: C: 37.91%, H: 1.36%, N: 1.58%; measured: C: 37.71%, H: 1.44%, N: 2.28%. A **molecular structure in solid state** was measured for this compound (table S4 and figure S26).

$[Ag(o,o\text{-}NC_5H_3F_2)_2][C_5(CF_3)_5]$ (7)

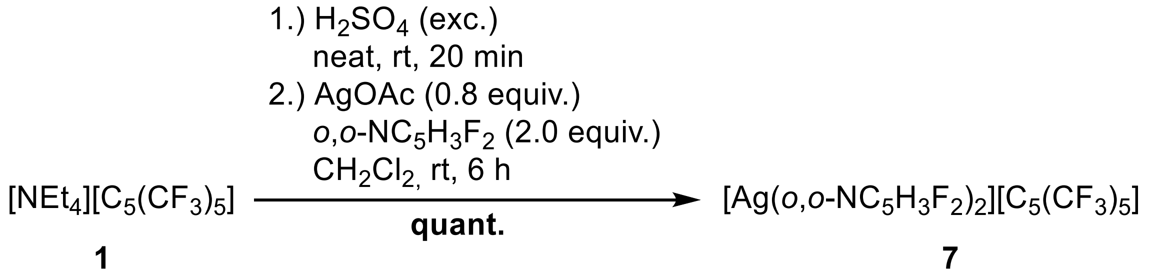

In a dried 10 mL Schlenk flask H_2SO_4 (conc., 2.0 mL, exc.) was placed under an argon atmosphere and cooled to −196 °C before $[NEt_4][C_5(CF_3)_5]$ (**1**, 0.10 g, 0.19 mmol, 1.0 equiv.) was added. Then the reaction mixture was warmed to rt under high vacuum and stirred at this temperature for 20 min. The volatiles were continuously trapped in a second dried 10 mL Schlenk flask cooled to −196 °C, containing AgOAc (25 mg, 0.15 mmol, 0.8 equiv.) and $o,o\text{-}NC_5H_3F_2$ (44 mg, 0.39 mmol, 2.0 equiv.) in anhydrous CH_2Cl_2 (2 mL). The second Schlenk flask was put under an atmosphere of argon, warmed to rt and stirred at this temperature for 6 h under the exclusion of light. The solvent was removed in high vacuum and the remaining solid was washed with anhydrous *n*-pentane (3 × 2 mL) and dried in high vacuum to give product $[Ag(o,o\text{-}NC_5H_3F_2)_2][C_5(CF_3)_5]$ (**7**, 89 mg, 0.15 mmol) as a colorless amorphous solid with a quantitative yield.

^1H NMR (400 MHz, CD_2Cl_2, rt) δ [ppm] = 8.14 (quint, $^3J_{H,H}$ = 7.9 Hz, 2H), 7.09 (d, $^3J_{H,H}$ = 8.1 Hz, 4H).[96] **^{19}F NMR** (377 MHz, CD_2Cl_2, rt) δ [ppm] = −52.1 (s, 15F), −65.1 (d, $^3J_{F,H}$ = 7.8 Hz, 4F). **$^{13}C\{^1H\}$ NMR** (101 MHz, CD_2Cl_2, rt) δ [ppm] = 160.9 (dd, $^1J_{C,F}$ = 255.1 Hz, $^3J_{C,F}$ = 10.6 Hz, 4C), 148.9 (t, $^3J_{C,F}$ = 8.7 Hz, 2C), 108.0–107.5 (m, 4C).[93] **FT-IR** (ATR) $\tilde{\nu}$ [cm^{-1}] = 3129 (w), 2805 (w), 1630 (s), 1588 (w), 1493 (m), 1467 (s), 1329 (w), 1276 (w), 1258 (m), 1200 (vs), 1108 (vs), 1028 (m), 1007 (vs), 883 (w), 796 (vs), 745 (s), 724 (m), 632 (vs), 573 (m). **HRMS** (ESI-TOF, negative) m/z for $[C_{10}F_{15}]^-$ calculated: 404.9760; measured: 404.9730. **EA** for $[C_{20}H_6F_{19}N_2Ag]$ calculated:

C: 32.33%, H: 0.81%, N: 3.77%; measured: C: 32.38%, H: 0.88%, N: 3.89%. A **molecular structure in solid state** was measured for this compound (table S5 and figure S27).

[H(*m*,*m*-$NC_5H_3F_2)_2][C_5(CF_3)_5]$ (8)

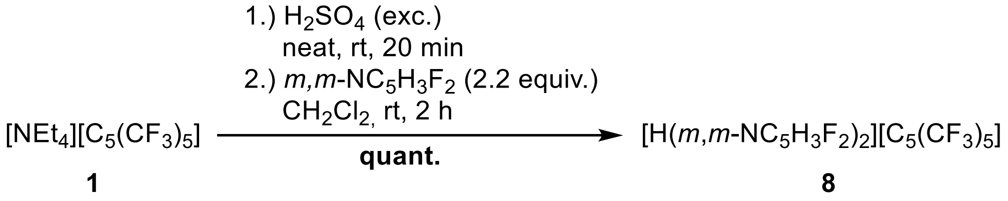

In a dried 10 mL Schlenk flask H_2SO_4 (conc., 2.0 mL, exc.) was placed under an argon atmosphere and cooled to −196 °C before $[NEt_4][C_5(CF_3)_5]$ (**1**, 0.10 g, 0.19 mmol, 1.0 equiv.) was added. Then the reaction mixture was warmed to rt under high vacuum and stirred at this temperature for 20 min. The volatiles were continuously trapped in a second dried 10 mL Schlenk flask cooled to −196 °C, containing *m,m*-$NC_5CH_3F_2$ (44 mg, 0.42 mmol, 2.2 equiv.) in anhydrous CH_2Cl_2 (2 mL). The second Schlenk flask was put under an atmosphere of argon, warmed to rt and stirred at this temperature for 2 h. The solvent was removed in high vacuum and the remaining solid was washed with anhydrous *n*-pentane (3 × 2 mL) and dried in high vacuum to give product [H(*m*,*m*-$NC_5H_3F_2)_2][C_5(CF_3)_5]$ (**8**, 121 mg, 0.19 mmol) as a colorless amorphous solid with a quantitative yield.

^{1}H NMR (400 MHz, CD_3CN, rt) δ [ppm] = 13.20 (s, 1H), 8.57 (s, 4H), 8.01 (s, 2H).[97] **^{19}F NMR** (377 MHz, CD_3CN, rt) δ [ppm] = −51.0 (s, 15F), −118.6 (d, $^3J_{F,H}$ = 9.0 Hz, 4F). **^{13}C{^{1}H} NMR** (176 MHz, CD_3CN, rt) δ [ppm] = 161.2 (dd, $^1J_{C,F}$ = 258.9 Hz, $^3J_{C,F}$ = 9.1 Hz, 4C), 132.4 (dd, $^2J_{C,F}$ = 28.2 Hz, $^4J_{C,F}$ = 6.4 Hz, 4C), 124.6 (q, $^1J_{C,F}$ = 270.9 Hz, 5C), 119.7 (t, $^2J_{C,F}$ = 21.8 Hz, 2C), 110.5 (m_c, 5C). **FT-IR** (ATR) ṽ [cm^{-1}] = 3125 (w), 3096 (w), 2730 (w), 2670 (w), 1567 (m), 1493 (m), 1469 (m), 1356 (w), 1321 (w), 1206 (vs), 1108 (vs), 983 (w), 905 (m), 871 (m), 845 (m), 801 (m), 738 (m), 671 (m), 633 (s), 532 (s). **HRMS** (ESI-TOF, negative) m/z for $[C_{10}F_{15}]^-$ calculated: 404.9760; measured: 404.9583. **EA** for $[C_{20}H_7F_{19}N_2]$ calculated: C: 37.76%, H: 1.11%, N: 4.40%; measured: C: 35.66%, H: 1.18%, N: 3.81%. A **molecular structure in solid state** was measured for this compound (table S6 and figure S28).

NMR Spectra

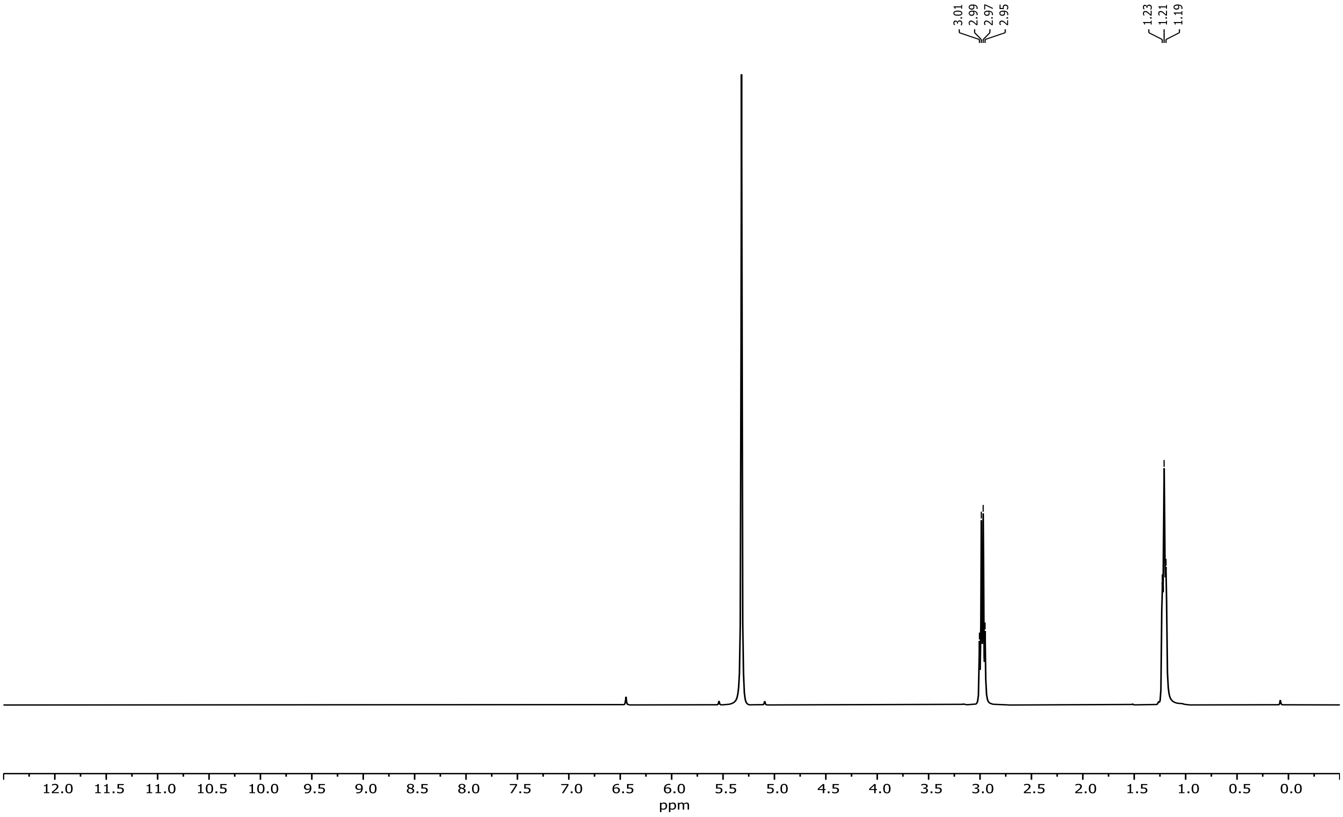

Figure S1. ^{1}H NMR (400 MHz, CD_2Cl_2, rt) spectrum of $[NEt_4][C_5(CF_3)_5]$ (**1**).

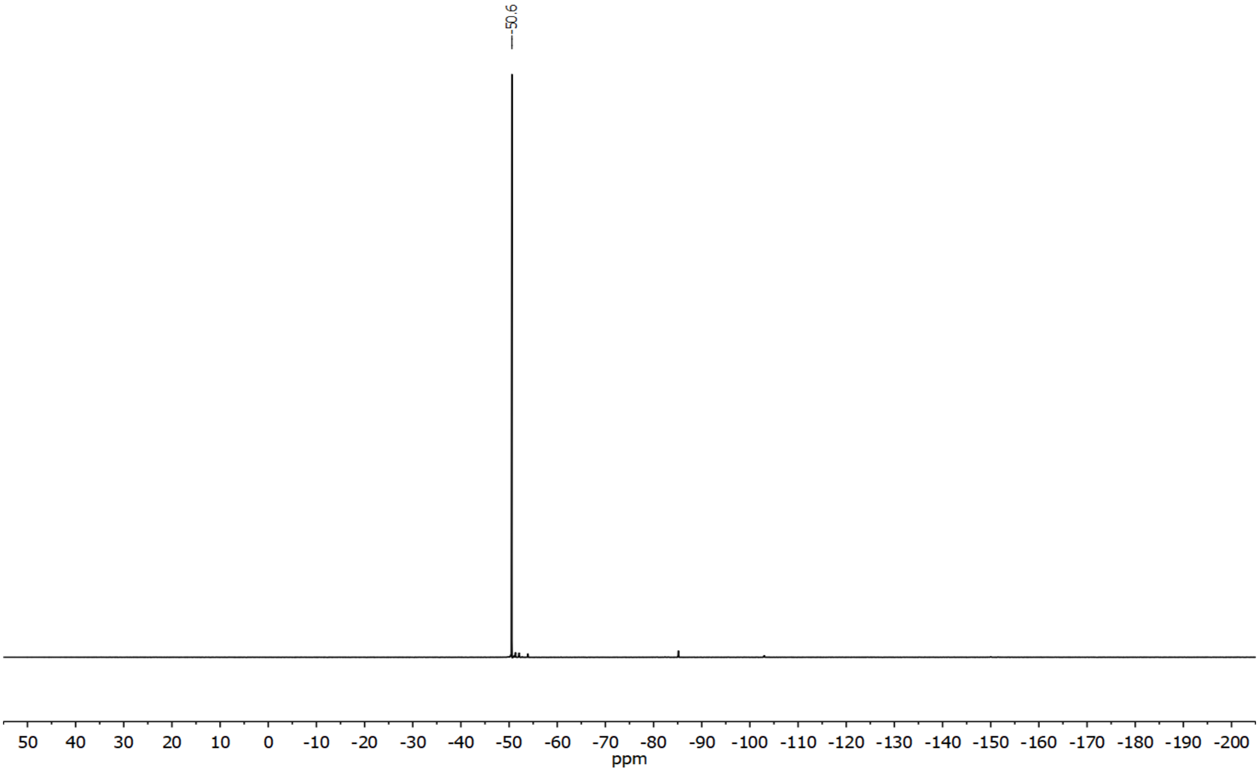

Figure S2. ^{19}F NMR (377 MHz, CD_2Cl_2, rt) spectrum of $[NEt_4][C_5(CF_3)_5]$ (**1**).

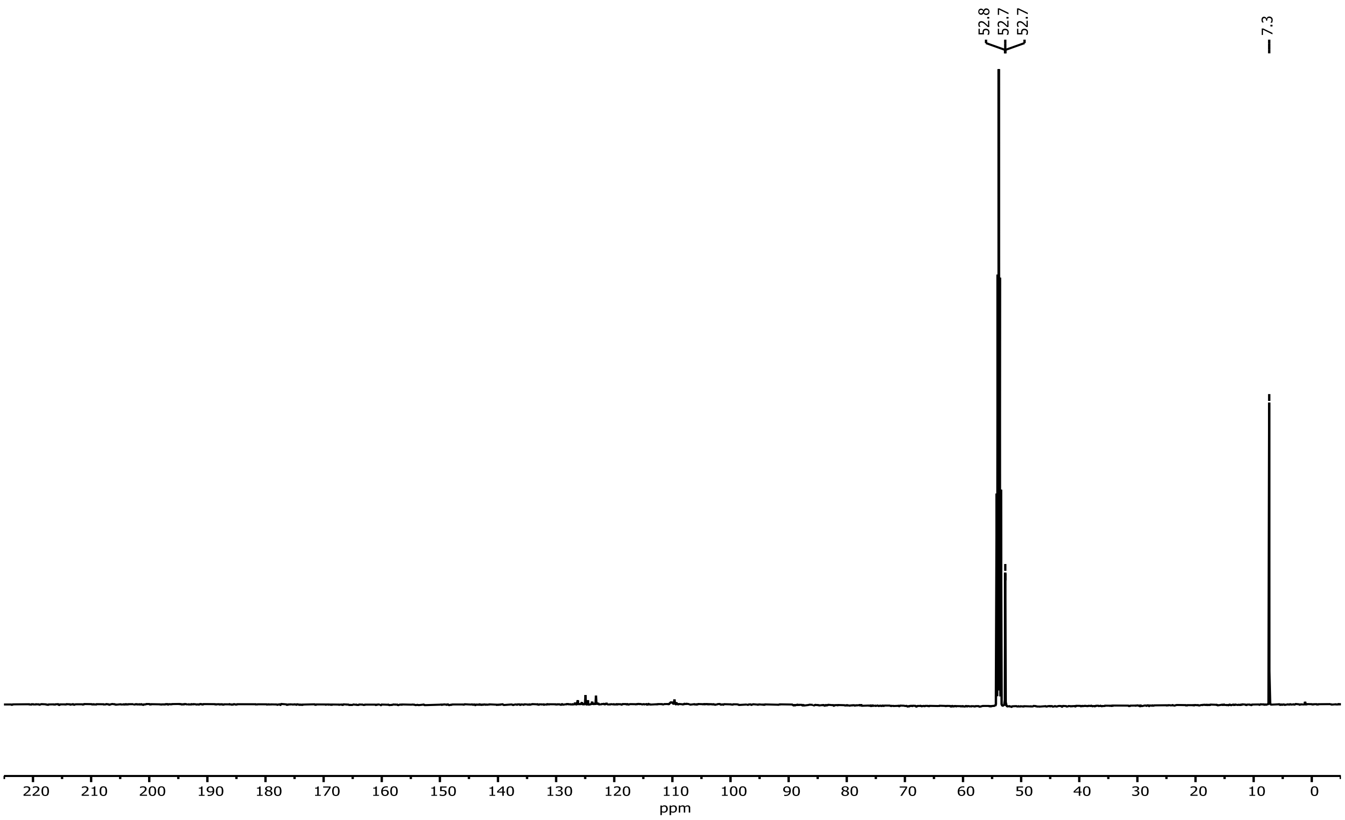

Figure S3. $^{13}C\{^1H\}$ NMR (151 MHz CD_2Cl_2, rt) spectrum of $[NEt_4][C_5(CF_3)_5]$ (**1**).

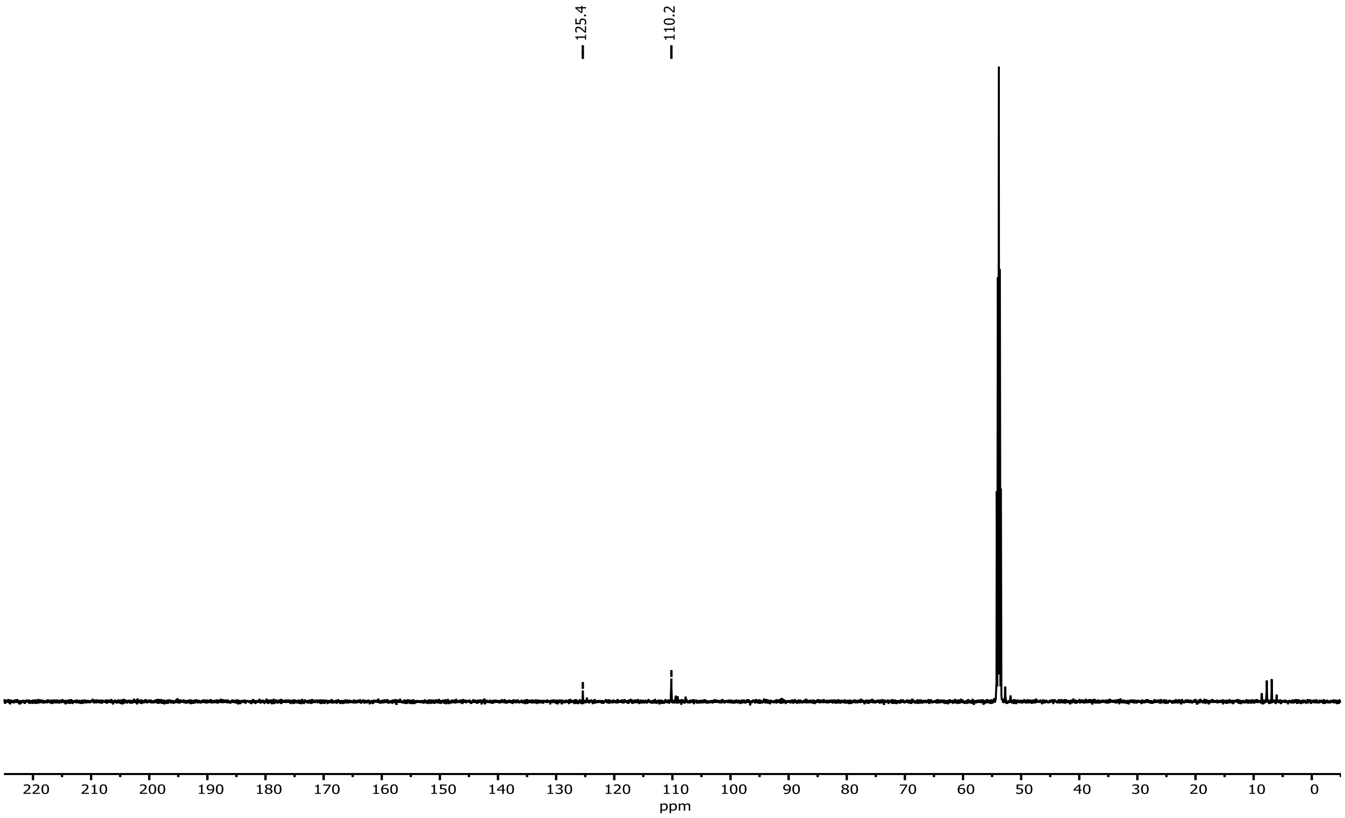

Figure S4. $^{13}C\{^{19}F\}$ NMR (151 MHz CD_2Cl_2, rt) spectrum of $[NEt_4][C_5(CF_3)_5]$ (**1**).

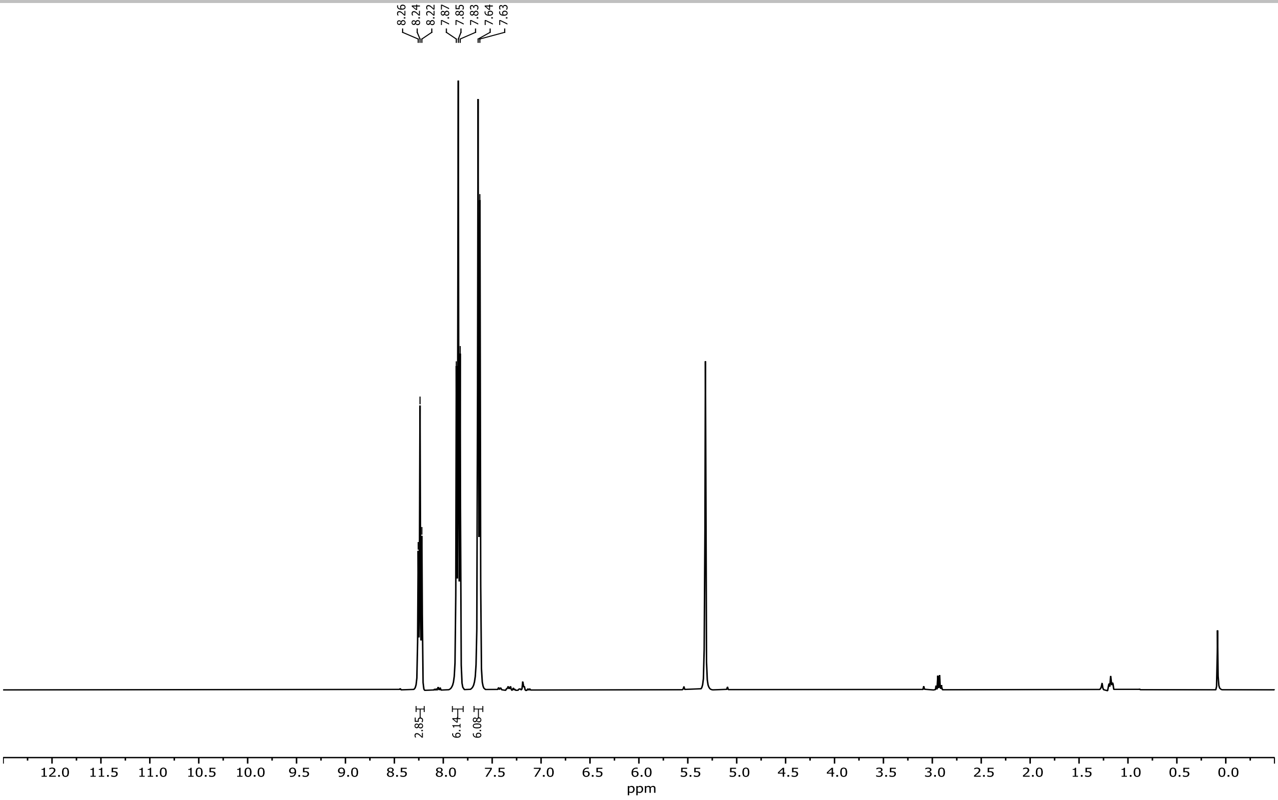

Figure S5. ^{1}H NMR (400 MHz, CD_2Cl_2, rt) spectrum of $[C(C_6H_5)_3][C_5(CF_3)_5]$ (**3**).[93]

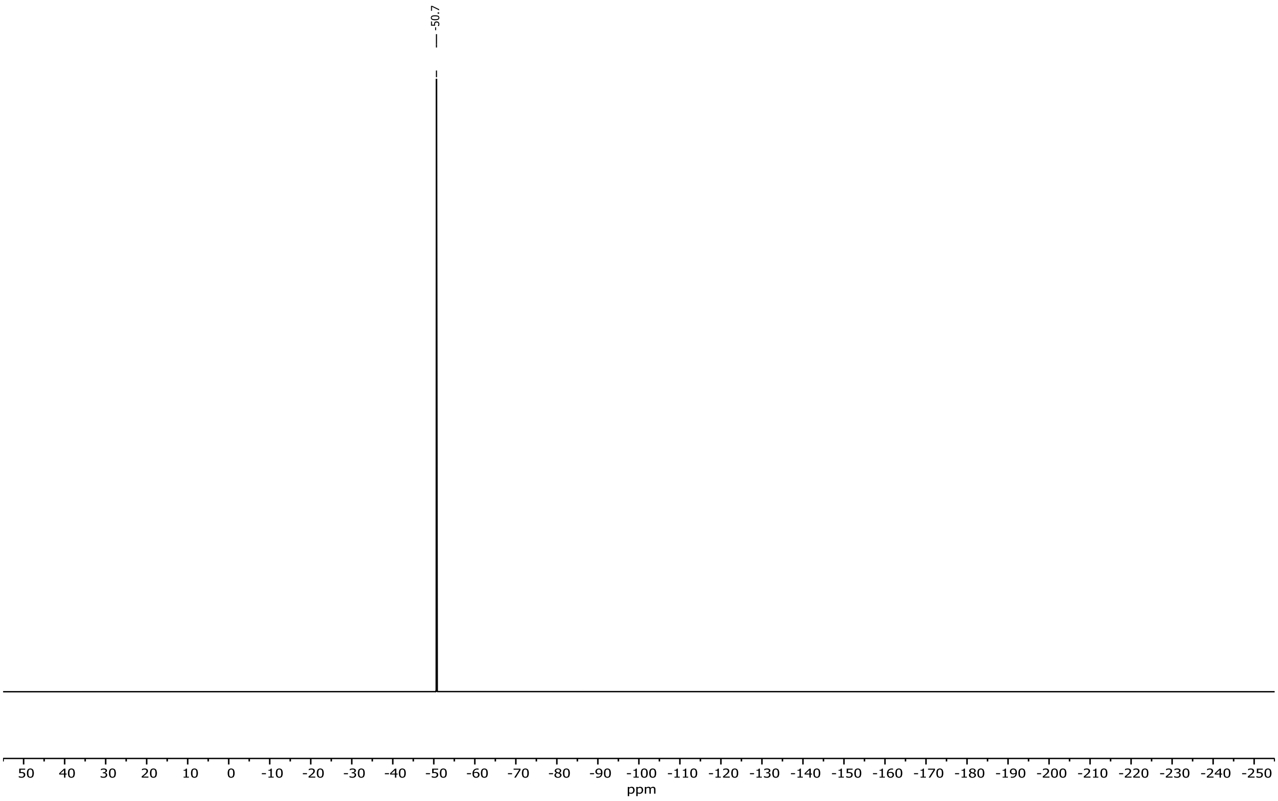

Figure S6. ^{19}F NMR (377 MHz, CD_2Cl_2, rt) spectrum of $[C(C_6H_5)_3][C_5(CF_3)_5]$ (**3**).

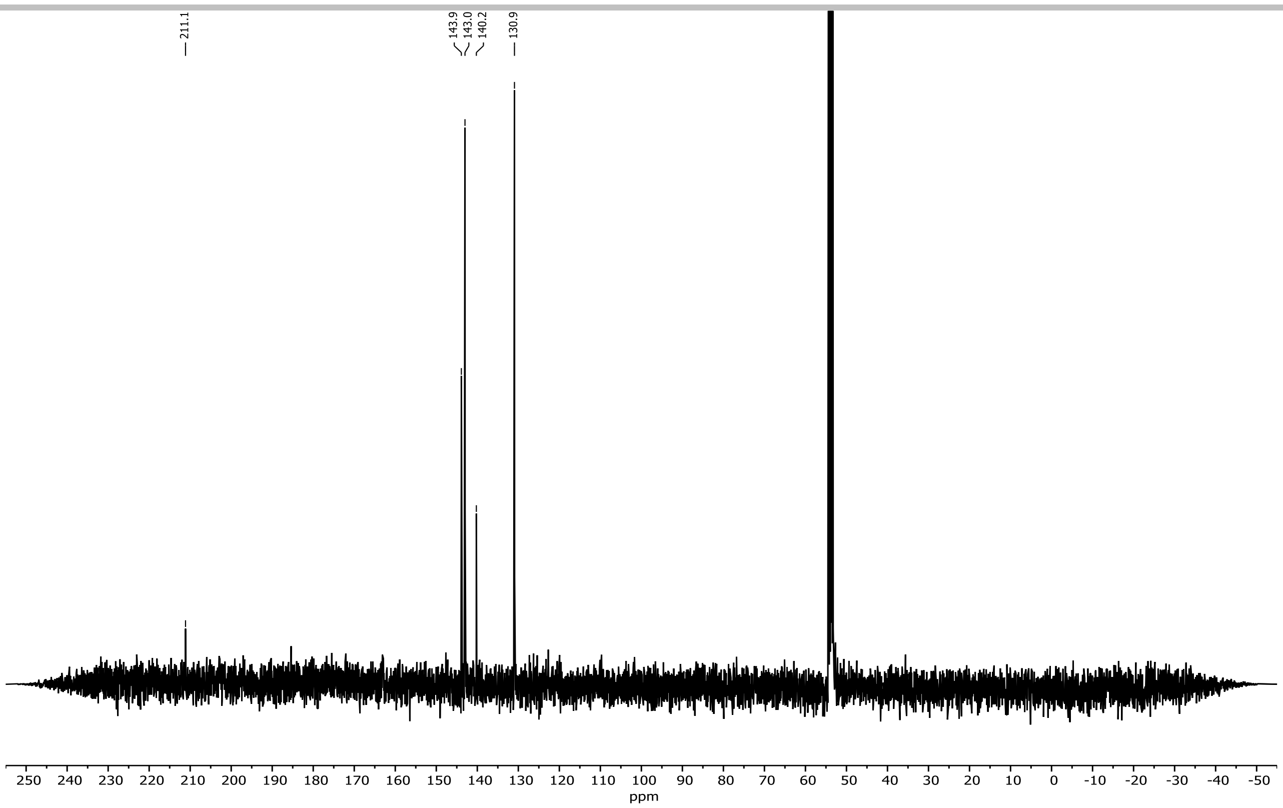

Figure S7. $^{13}C\{^{1}H\}$ NMR (101 MHz, CD_2Cl_2, rt) spectrum of $[C(C_6H_5)_3][C_5(CF_3)_5]$ (**3**).

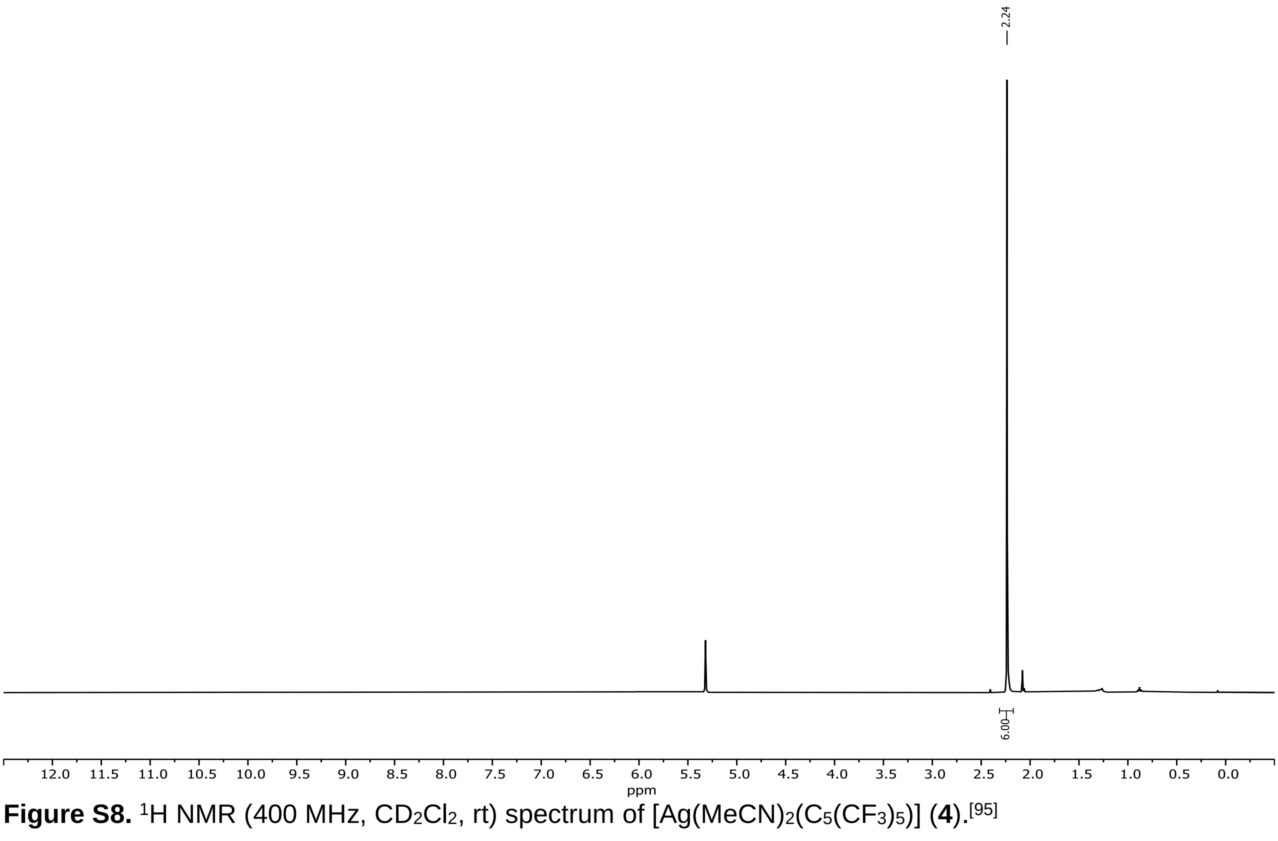

Figure S8. ^{1}H NMR (400 MHz, CD_2Cl_2, rt) spectrum of $[Ag(MeCN)_2(C_5(CF_3)_5)]$ (**4**).[95]

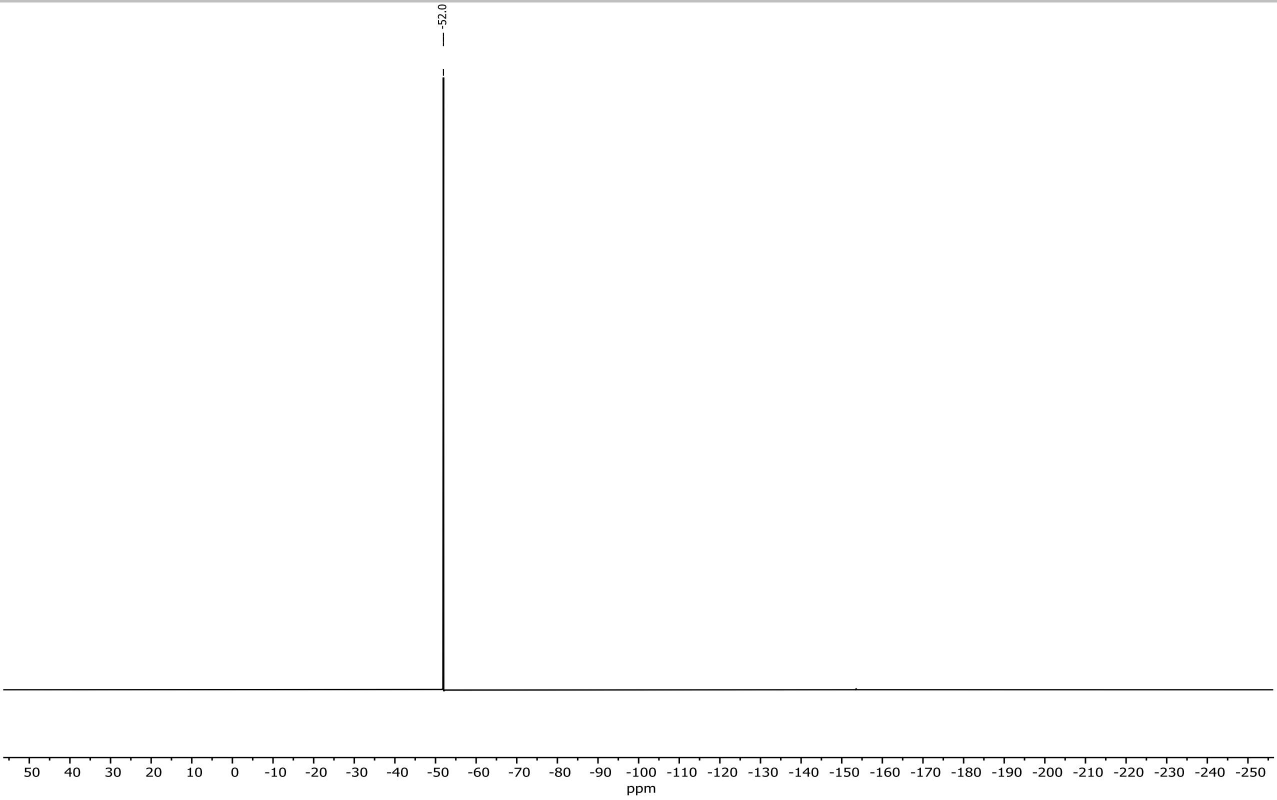

Figure S9. ^{19}F NMR (377 MHz, CD_2Cl_2, rt) spectrum of $[Ag(MeCN)_2(C_5(CF_3)_5)]$ (**4**).

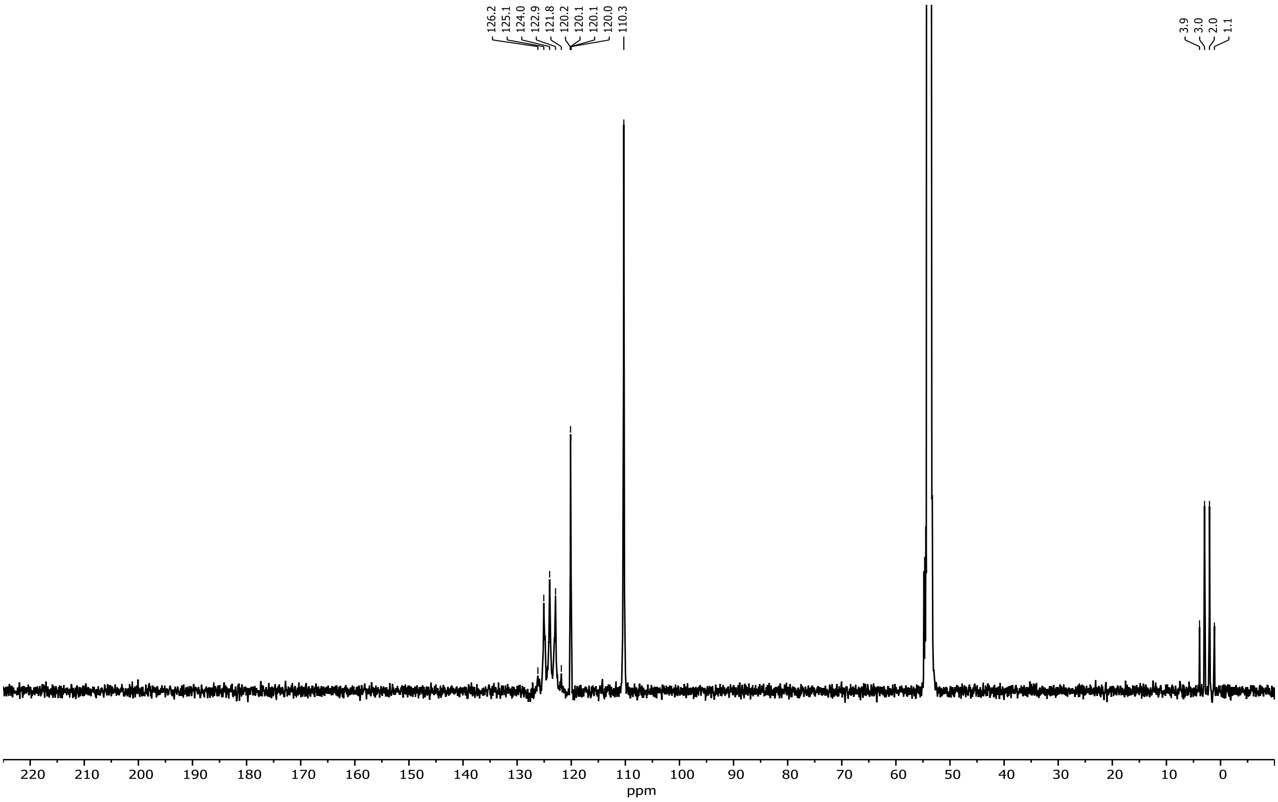

Figure S10. $^{13}C\{^{19}F\}$ NMR (151 MHz, CD_2Cl_2, rt) spectrum of $[Ag(MeCN)_2(C_5(CF_3)_5)]$ (**4**).

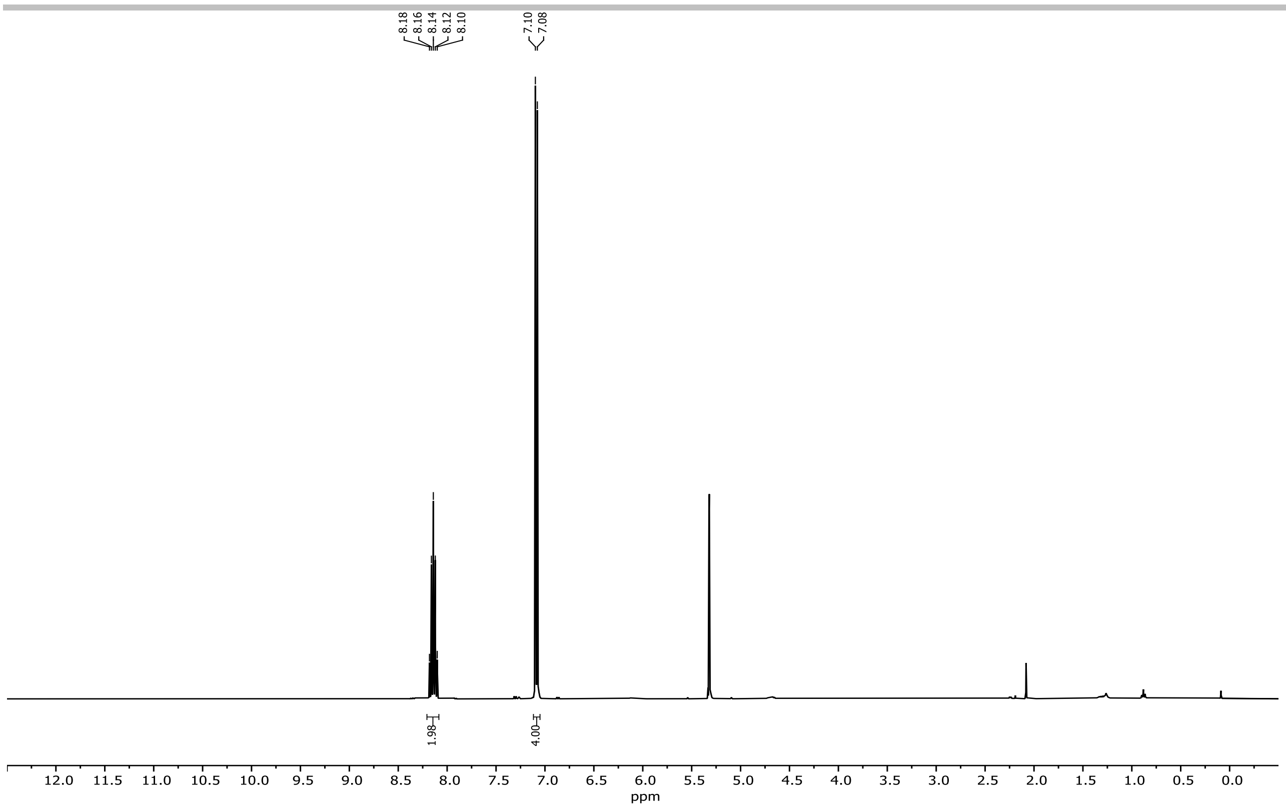

Figure S11. ^{1}H NMR (400 MHz CD_2Cl_2, rt) spectrum of [Ag(*o*,*o*-$NC_5H_3F_2$)$_2$][$C_5(CF_3)_5$] (**7**).[96]

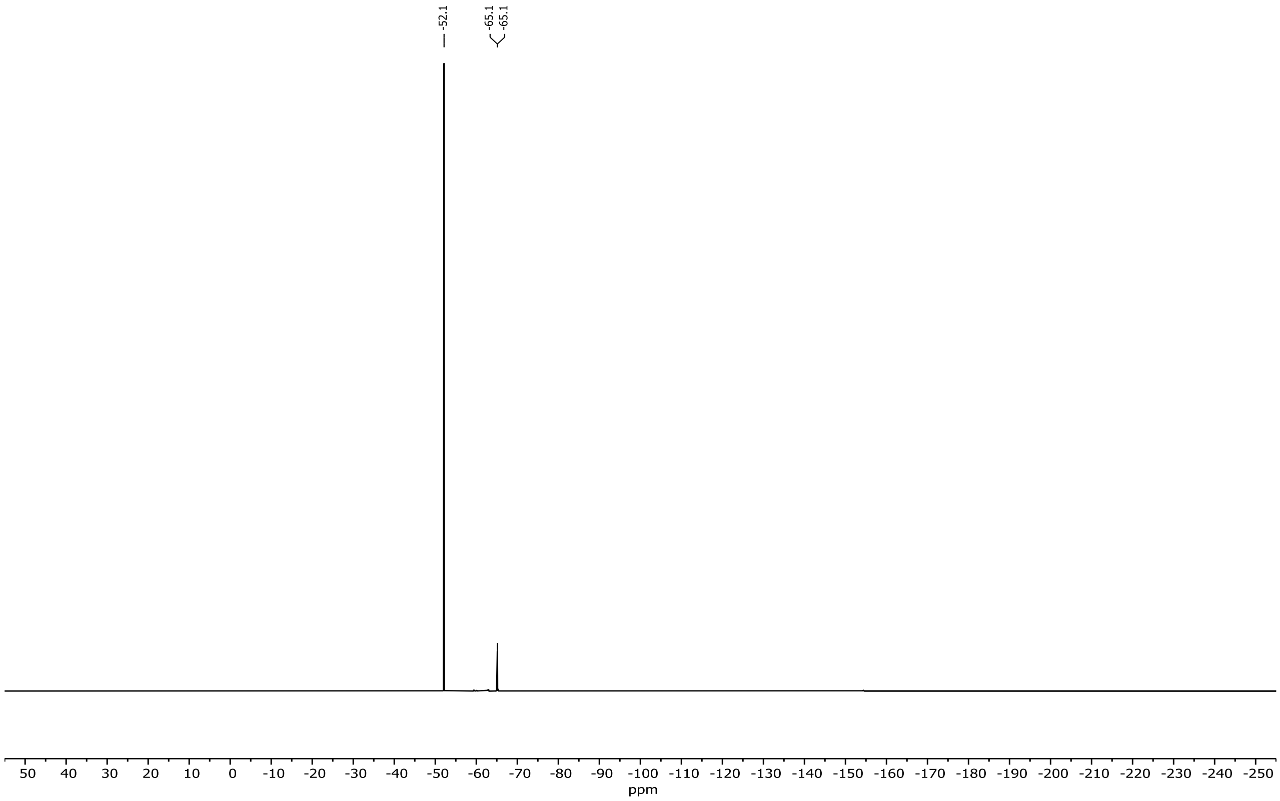

Figure S12. ^{19}F NMR (377 MHz, CD_2Cl_2, rt) spectrum of [Ag(o,o-$NC_5H_3F_2$)$_2$][$C_5(CF_3)_5$] (**7**).

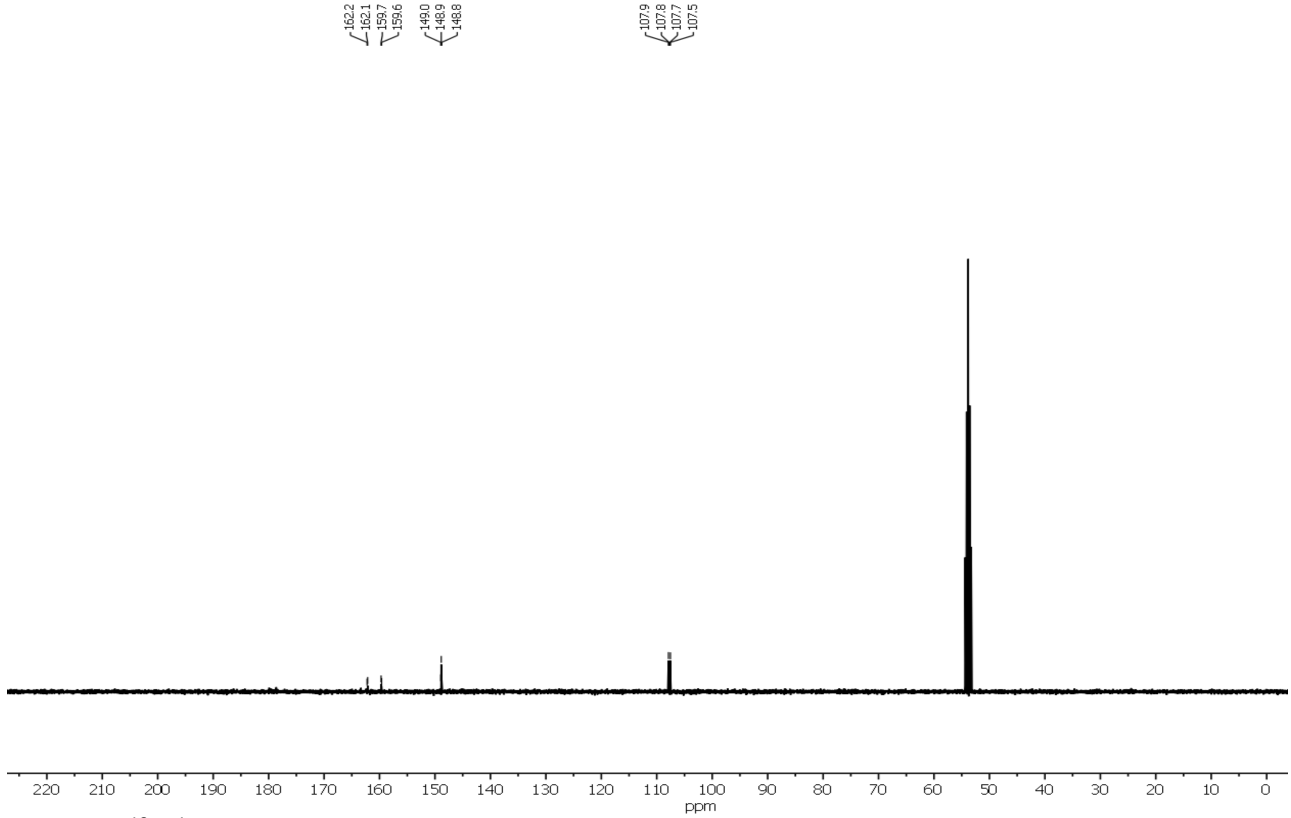

Figure S13. $^{13}C\{^{1}H\}$ NMR (101 MHz, CD_2Cl_2, rt) spectrum of [Ag(o,o-$NC_5H_3F_2)_2$][$C_5(CF_3)_5$] (**7**).

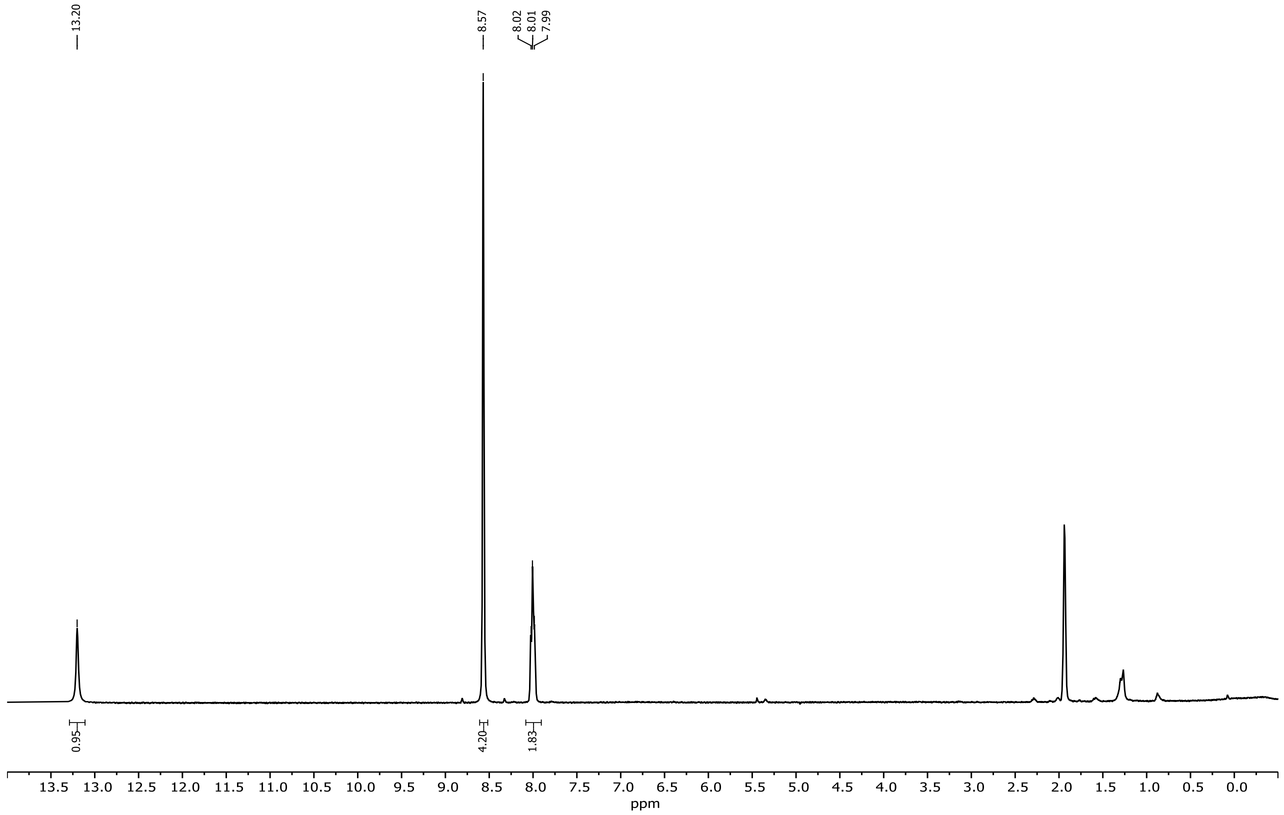

Figure S14. ^{1}H NMR (400 MHz, CD_3CN, rt) spectrum of [H(*m*,*m*-$NC_5H_3F_2)_2$][$C_5(CF_3)_5$] (**8**).[97]

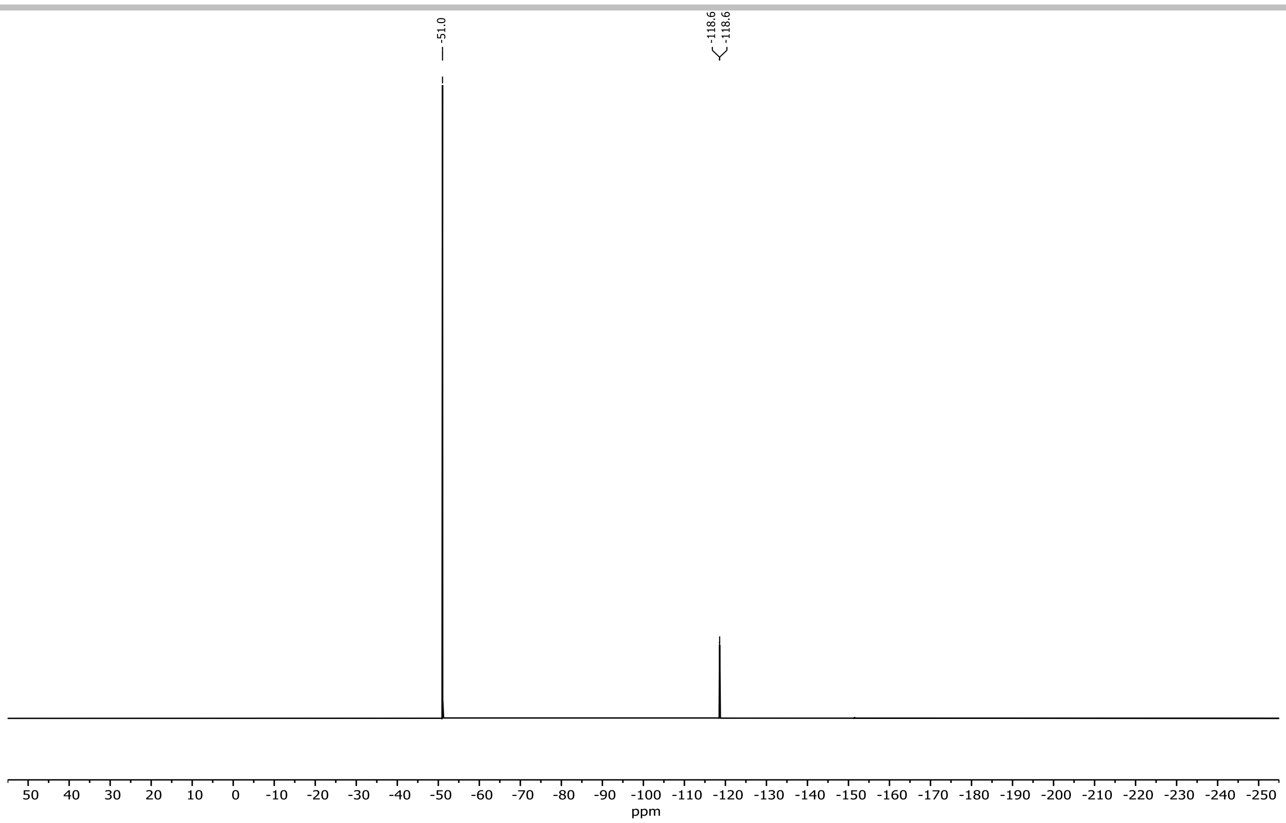

Figure S15. ^{19}F NMR (377 MHz, CD_3CN, rt) spectrum of [H(*m*,*m*-$NC_5H_3F_2$)$_2$][$C_5(CF_3)_5$] (**8**).

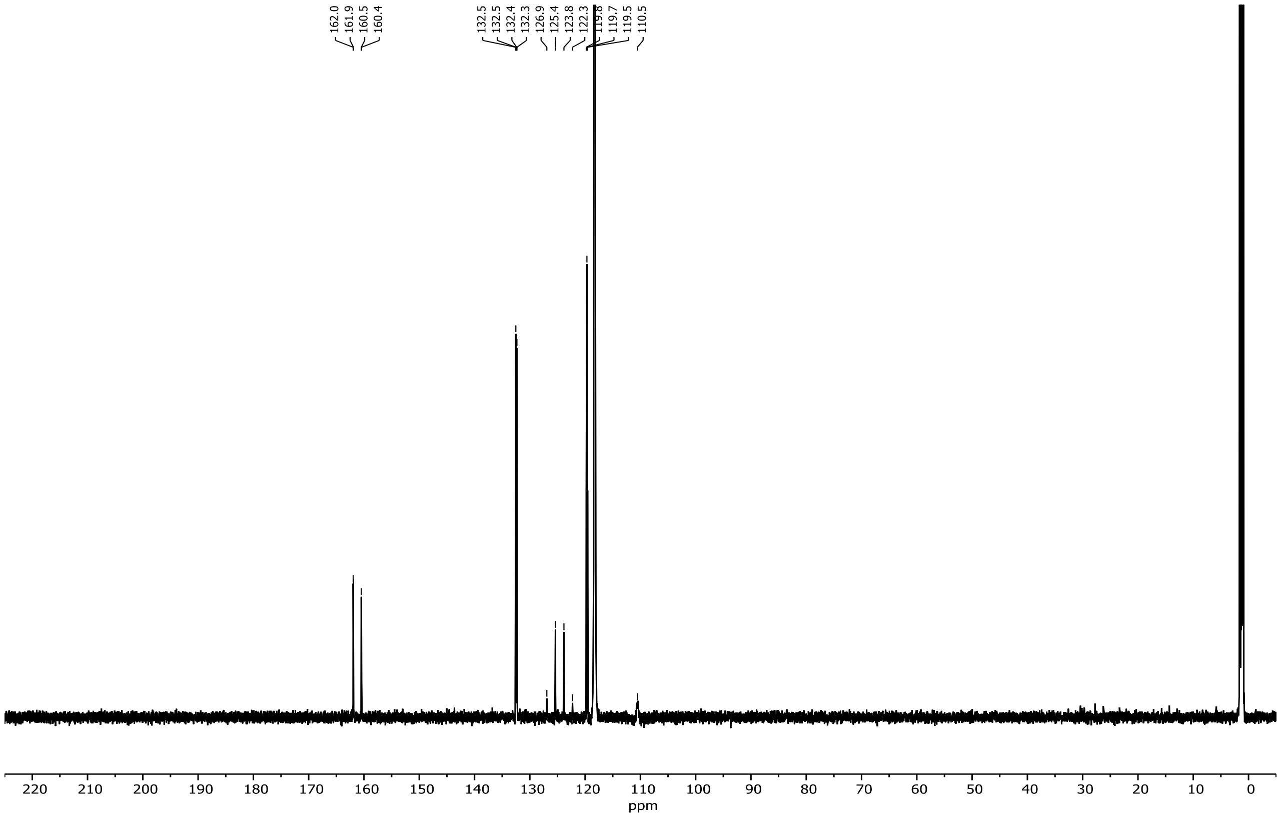

Figure S16. ^{13}C{^{1}H} NMR (101 MHz, CD_3CN, rt) spectrum of [H(*m*,*m*-$NC_5H_3F_2$)$_2$][$C_5(CF_3)_5$] (**8**).

IR Spectra

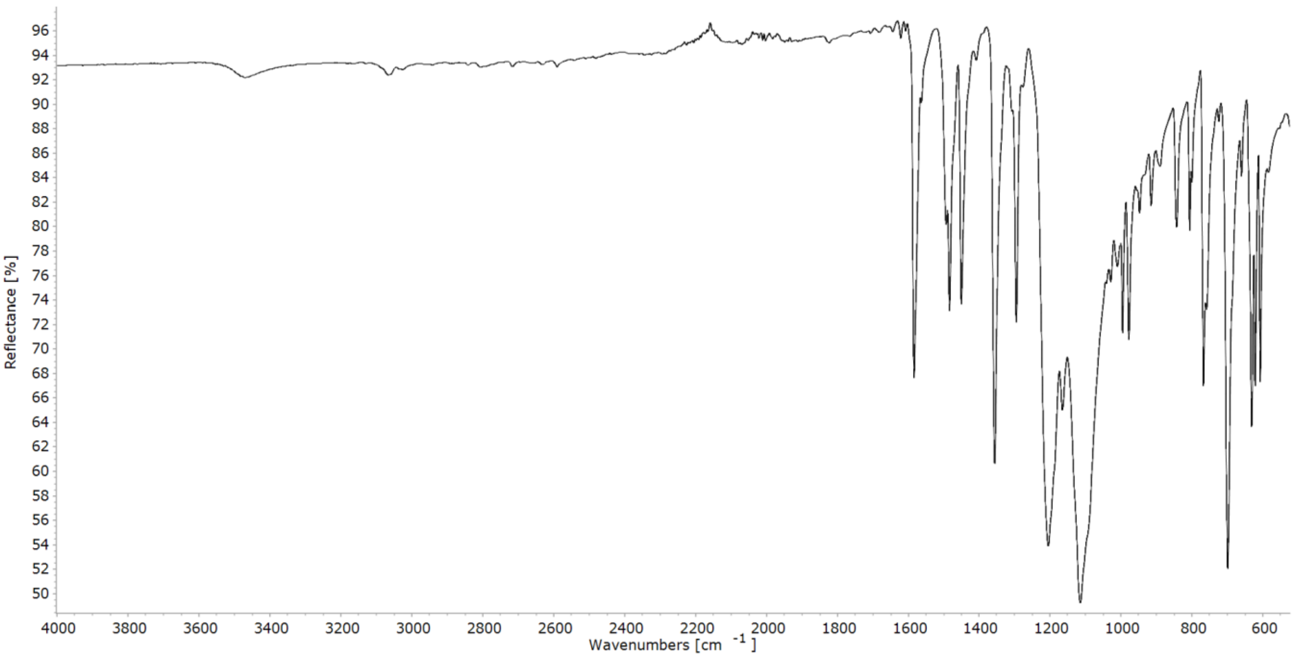

Figure S17. IR (ATR, rt) spectrum of $[C(C_6H_5)_3][C_5(CF_3)_5]$ (**3**).

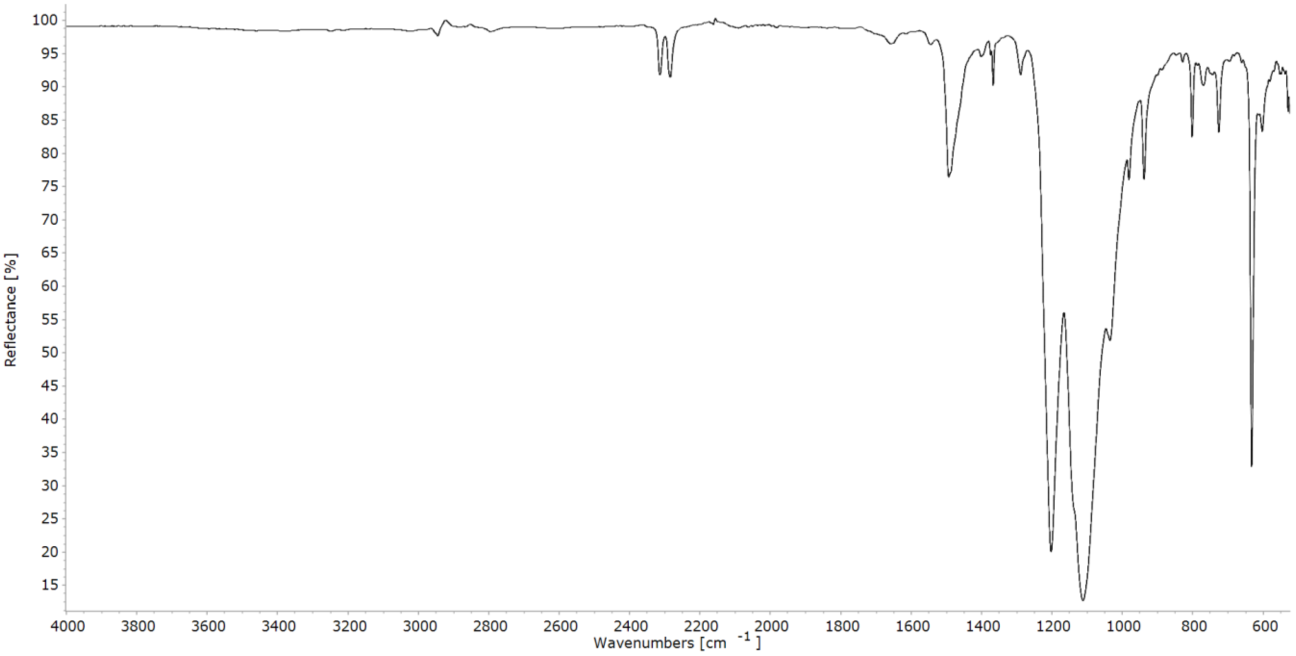

Figure S18. IR (ATR, rt) spectrum of $[Ag(MeCN)_2(C_5(CF_3)_5)]$ (**4**).

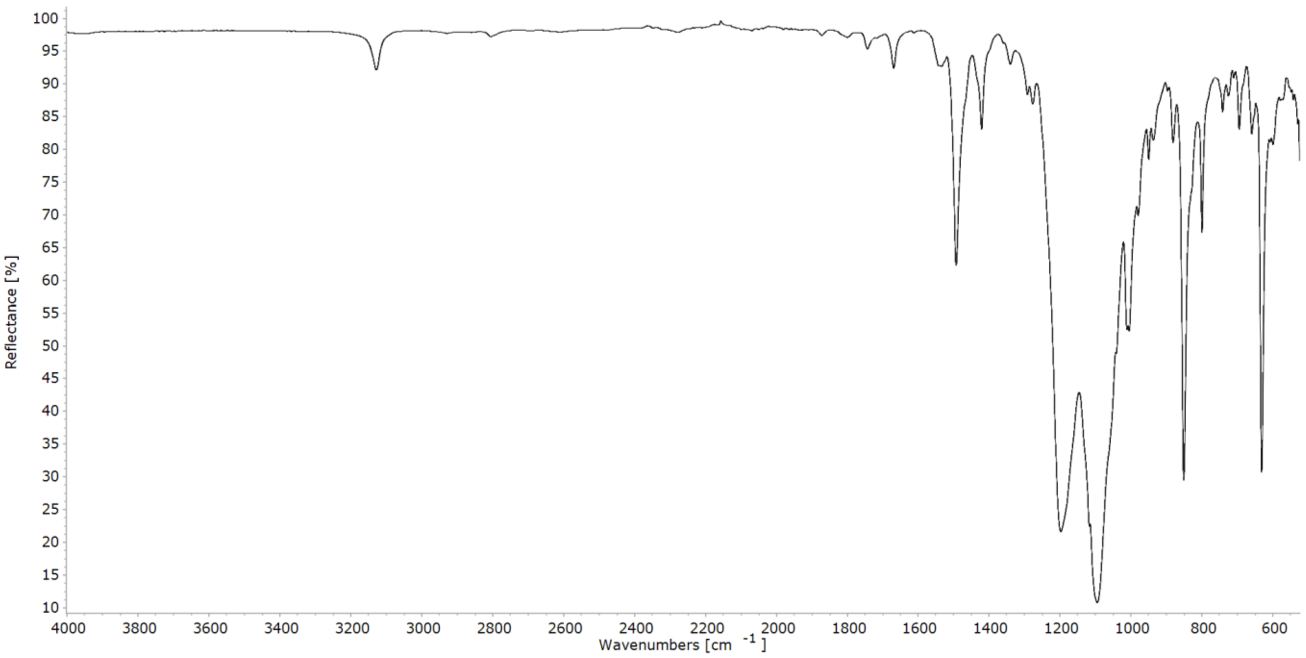

Figure S19. IR (ATR, rt) spectrum of $[Fe(C_5H_5)_2][C_5(CF_3)_5]$ (**5**).

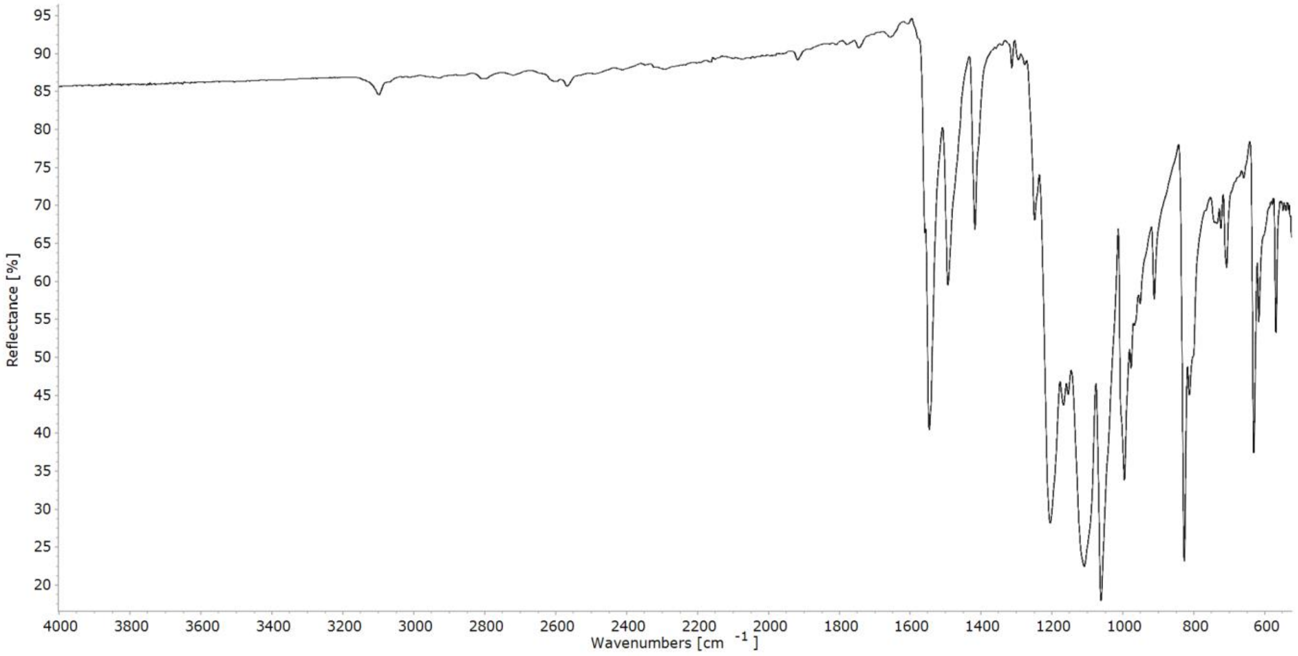

Figure S20. IR (ATR, rt) spectrum of $[N(p\text{-}C_6H_4Br)_3][C_5(CF_3)_5]$ (**6**).

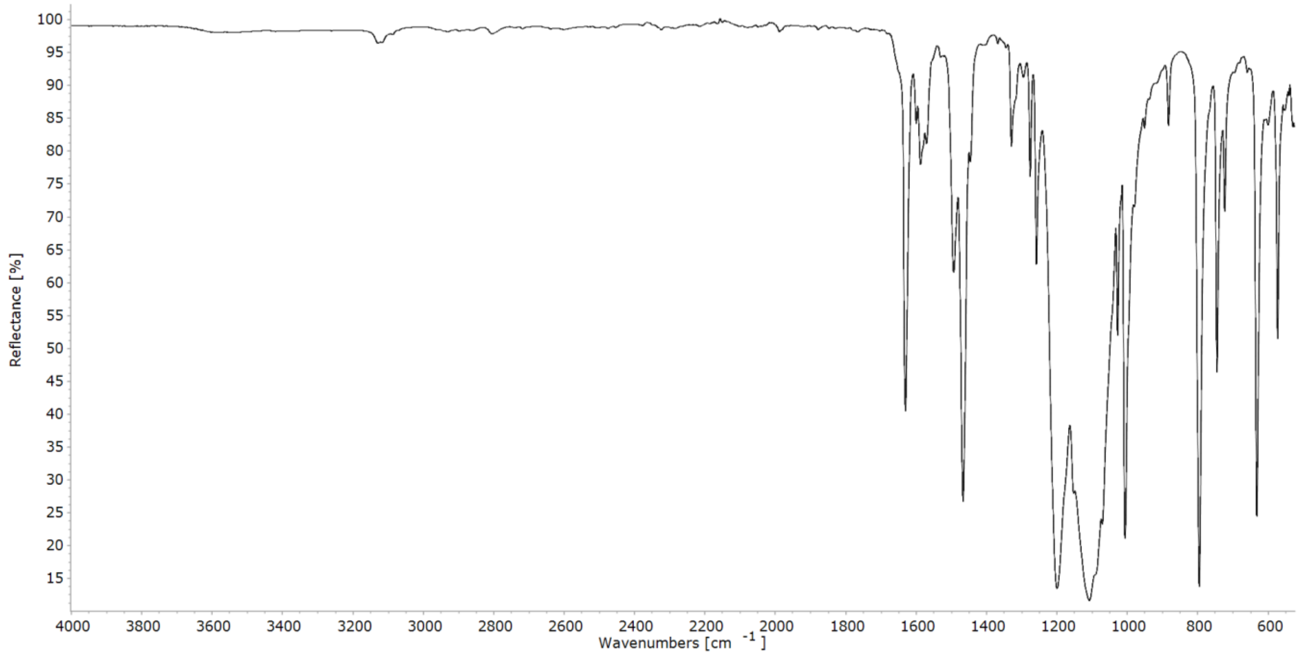

Figure S21. IR (ATR, rt) spectrum of [Ag(*o*,*o*-$NC_5H_3F_2$)$_2$][$C_5(CF_3)_5$] (**7**).

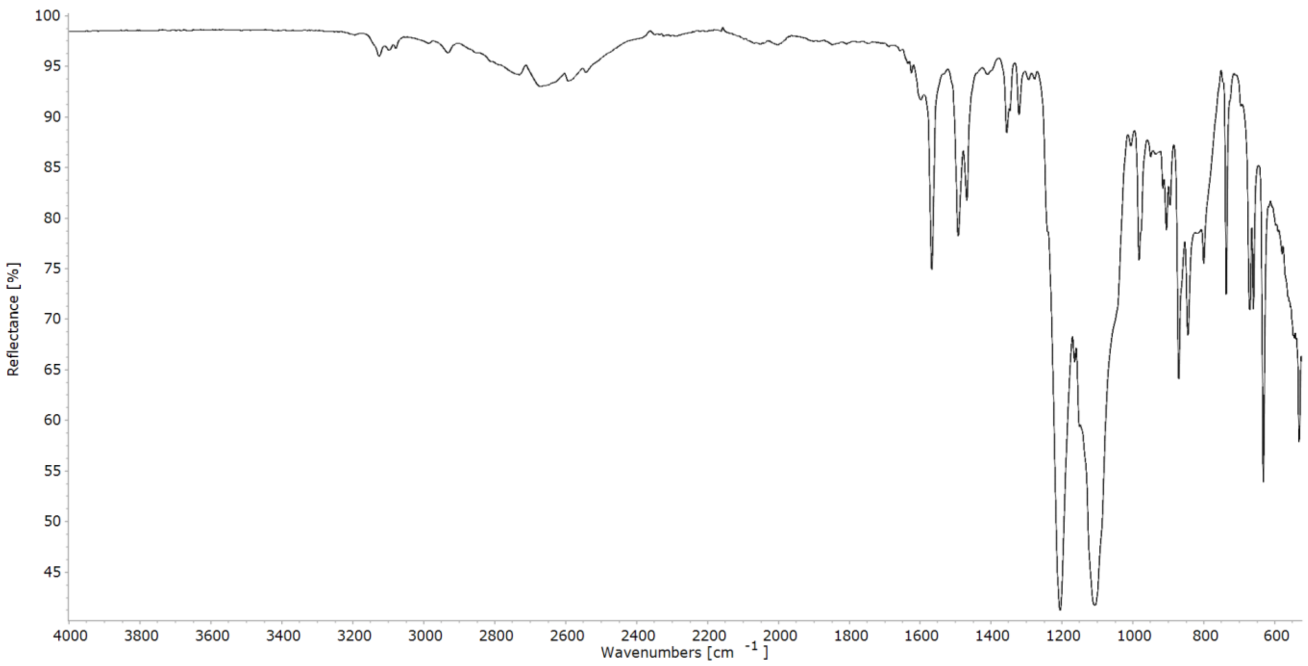

Figure S22. IR (ATR, rt) spectrum of [H(*m*,*m*-$NC_5H_3F_2$)$_2$][$C_5(CF_3)_5$] (**8**).

Crystallographic Data

Table S1. Crystallographic data of $[C(C_6H_5)_3][C_5(CF_3)_5]$ (**3**).

Identification code	2426566
Empirical formula	$C_{29}H_{15}F_{15}$
Formula weight	648.41
Temperature/K	100
Crystal system	monoclinic
Space group	$P2_1/n$
a/Å	10.2431(7)
b/Å	17.2823(10)
c/Å	14.5424(9)
α/°	90
β/°	95.135(3)
γ/°	90
Volume/Å^3	2564.0(3)
Z	4
ρ_{calc}g/cm^3	1.680
μ/mm^{-1}	0.174
F(000)	1296.0
Crystal size/mm^3	0.1 × 0.1 × 0.1
Radiation	MoKα (λ = 0.71073)
2Θ range for data collection/°	4.636 to 50.814
Index ranges	-12 ≤ h ≤ 12, -20 ≤ k ≤ 20, -17 ≤ l ≤ 17
Reflections collected	48331
Independent reflections	4716 [R_{int} = 0.0500, R_{sigma} = 0.0229]
Data/restraints/parameters	4716/0/397
Goodness-of-fit on F^2	1.120
Final R indexes [I>=2σ (I)]	R_1 = 0.0440, wR_2 = 0.0980
Final R indexes [all data]	R_1 = 0.0527, wR_2 = 0.1022
Largest diff. peak/hole / e Å^{-3}	0.34/-0.22

Table S2. Crystallographic data of [Ag(MeCN)$_2$(C$_5$(CF$_3$)$_5$)] (**4**).

Identification code	2426567
Empirical formula	$C_{14}H_6AgF_{15}N_2$
Formula weight	595.08
Temperature/K	100
Crystal system	monoclinic
Space group	$P2_1/n$
a/Å	9.0675(8)
b/Å	14.8720(15)
c/Å	13.3849(12)
α/°	90
β/°	90.243(4)
γ/°	90
Volume/Å^3	1805.0(3)
Z	24
ρ_{calc}g/cm^3	2.190
μ/mm^{-1}	1.271
F(000)	1144.0
Crystal size/mm^3	0.1 × 0.1 × 0.1
Radiation	MoKα (λ = 0.71073)
2Θ range for data collection/°	5.262 to 61.054
Index ranges	-12 ≤ h ≤ 12, -19 ≤ k ≤ 21, -19 ≤ l ≤ 18
Reflections collected	45304
Independent reflections	5489 [R_{int} = 0.0290, R_{sigma} = 0.0165]
Data/restraints/parameters	5489/0/291
Goodness-of-fit on F^2	1.041
Final R indexes [I>=2σ (I)]	R_1 = 0.0489, wR_2 = 0.1255
Final R indexes [all data]	R_1 = 0.0522, wR_2 = 0.1280
Largest diff. peak/hole / e Å^{-3}	2.36/-0.94

Table S3. Crystallographic data of $[Fe(C_5H_5)_2][C_5(CF_3)_5]$ (**5**).

Identification code	2426568
Empirical formula	$C_{20}H_{10}F_{15}Fe$
Formula weight	591.13
Temperature/K	100.00
Crystal system	triclinic
Space group	$P\bar{1}$
a/Å	10.5019(3)
b/Å	14.4623(5)
c/Å	15.1556(6)
α/°	112.9090(10)
β/°	96.8350(10)
γ/°	99.3770(10)
Volume/Å^3	2049.38(12)
Z	4
ρ_{calc}g/cm^3	1.916
μ/mm^{-1}	0.878
F(000)	1164.0
Crystal size/mm^3	0.1 × 0.1 × 0.1
Radiation	MoKα (λ = 0.71073)
2Θ range for data collection/°	4.012 to 55.004
Index ranges	-13 ≤ h ≤ 13, -18 ≤ k ≤ 18, -19 ≤ l ≤ 19
Reflections collected	90102
Independent reflections	9414 [R_{int} = 0.0390, R_{sigma} = 0.0184]
Data/restraints/parameters	9414/0/649
Goodness-of-fit on F^2	1.048
Final R indexes [I>=2σ (I)]	R_1 = 0.0467, wR_2 = 0.1242
Final R indexes [all data]	R_1 = 0.0544, wR_2 = 0.1298
Largest diff. peak/hole / e Å^{-3}	1.71/-0.57

Table S4. Crystallographic data of [N(*p*-$C_6H_4Br)_3$][$C_5(CF_3)_5$] (**6**) · SO_2ClF

Identification code	2426569
Empirical formula	$C_{28}H_{12}Br_3ClF_{16}NO_2S$
Formula weight	1005.63
Temperature/K	100
Crystal system	orthorhombic
Space group	$Pna2_1$
a/Å	19.070(6)
b/Å	8.905(3)
c/Å	19.961(6)
α/°	90
β/°	90
γ/°	90
Volume/Å^3	3390(2)
Z	4
ρ_{calc}g/cm^3	1.970
μ/mm^{-1}	3.829
F(000)	1940.0
Crystal size/mm^3	0.1 × 0.1 × 0.1
Radiation	MoKα (λ = 0.71073)
2Θ range for data collection/°	4.08 to 50.99
Index ranges	-22 ≤ h ≤ 22, -10 ≤ k ≤ 9, -23 ≤ l ≤ 21
Reflections collected	19432
Independent reflections	5970 [R_{int} = 0.0788, R_{sigma} = 0.0910]
Data/restraints/parameters	5970/1/487
Goodness-of-fit on F^2	1.050
Final R indexes [I>=2σ (I)]	R_1 = 0.0501, wR_2 = 0.0957
Final R indexes [all data]	R_1 = 0.0740, wR_2 = 0.1037
Largest diff. peak/hole / e Å^{-3}	1.06/-0.67
Flack parameter	0.031(8)

Table S5. Crystallographic data of [Ag(*o*,*o*-$NC_5H_3F_2)_2$][$C_5(CF_3)_5$] (**7**).[98]

Identification code	2426570
Empirical formula	$C_{20}H_6AgF_{19}N_2$
Formula weight	743.14
Temperature/K	100.0
Crystal system	triclinic
Space group	$P\bar{1}$
a/Å	9.2859(2)
b/Å	14.2322(4)
c/Å	15.0815(4)
α/°	63.1610(10)
β/°	85.5230(10)
γ/°	78.1150(10)
Volume/Å^3	1740.07(8)
Z	3
ρ_{calc}g/cm^3	2.128
μ/mm^{-1}	1.036
F(000)	1074.0
Crystal size/mm^3	0.1 × 0.1 × 0.1
Radiation	MoKα (λ = 0.71073)
2Θ range for data collection/°	5.016 to 52.762
Index ranges	-11 ≤ h ≤ 11, -17 ≤ k ≤ 17, -18 ≤ l ≤ 18
Reflections collected	48175
Independent reflections	7061 [R_{int} = 0.0293, R_{sigma} = 0.0163]
Data/restraints/parameters	7061/0/536
Goodness-of-fit on F^2	1.031
Final R indexes [I>=2σ (I)]	R_1 = 0.0951, wR_2 = 0.2621
Final R indexes [all data]	R_1 = 0.1002, wR_2 = 0.2677
Largest diff. peak/hole / e Å^{-3}	1.98/-1.84

Table S6. Crystallographic data of $[H(m,m\text{-}NC_5H_3F_2)_2][C_5(CF_3)_5]$ (**8**).[98]

Identification code	2426571
Empirical formula	$C_{20}H_7F_{19}N_2$
Formula weight	636.28
Temperature/K	100
Crystal system	monoclinic
Space group	$P2_1/c$
a/Å	9.1677(2)
b/Å	12.7741(4)
c/Å	19.1797(6)
α/°	90
β/°	103.0240(10)
γ/°	90
Volume/Å^3	2188.34(11)
Z	4
ρ_{calc}g/cm^3	1.931
μ/mm^{-1}	0.229
F(000)	1248.0
Crystal size/mm^3	0.1 × 0.1 × 0.1
Radiation	MoKα (λ = 0.71073)
2Θ range for data collection/°	3.862 to 50.276
Index ranges	-10 ≤ h ≤ 10, -15 ≤ k ≤ 15, -22 ≤ l ≤ 22
Reflections collected	34676
Independent reflections	3909 [R_{int} = 0.0376, R_{sigma} = 0.0175]
Data/restraints/parameters	3909/0/429
Goodness-of-fit on F^2	1.047
Final R indexes [I>=2σ (I)]	R_1 = 0.0900, wR_2 = 0.2348
Final R indexes [all data]	R_1 = 0.1077, wR_2 = 0.2610
Largest diff. peak/hole / e Å^{-3}	0.89/-0.69

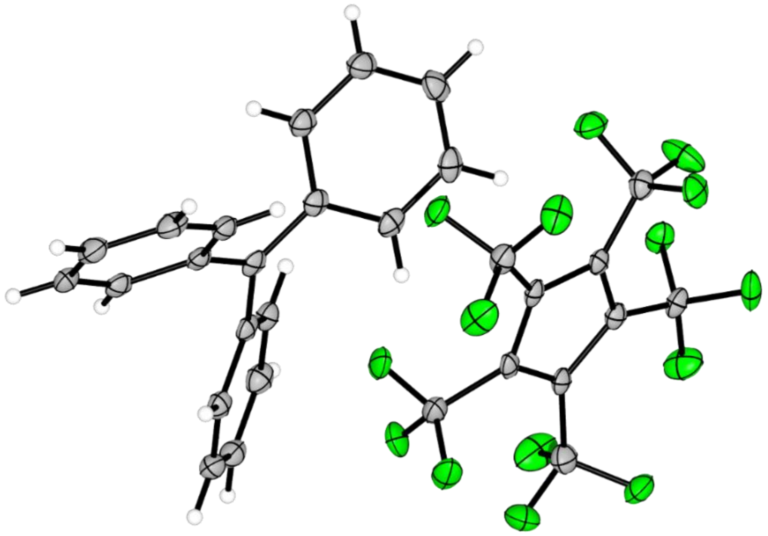

Figure S23. Molecular structure in solid state of $[C(C_6H_5)_3][C_5(CF_3)_5]$ (**3**). Ellipsoids are depicted with 50% probability level. Color code: white-hydrogen, grey-carbon, green-fluorine.

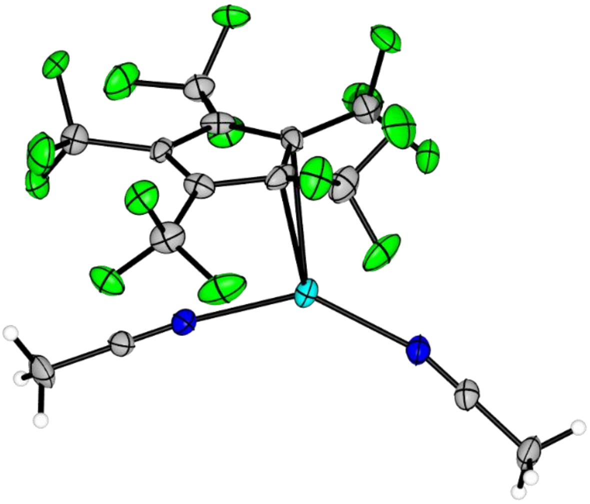

Figure S24. Molecular structure in solid state of $[Ag(MeCN)_2(C_5(CF_3)_5)]$ (**4**). Ellipsoids are depicted with 50% probability level. Color code: white-hydrogen, grey-carbon, green-fluorine, light blue-silver, deep blue-nitrogen.

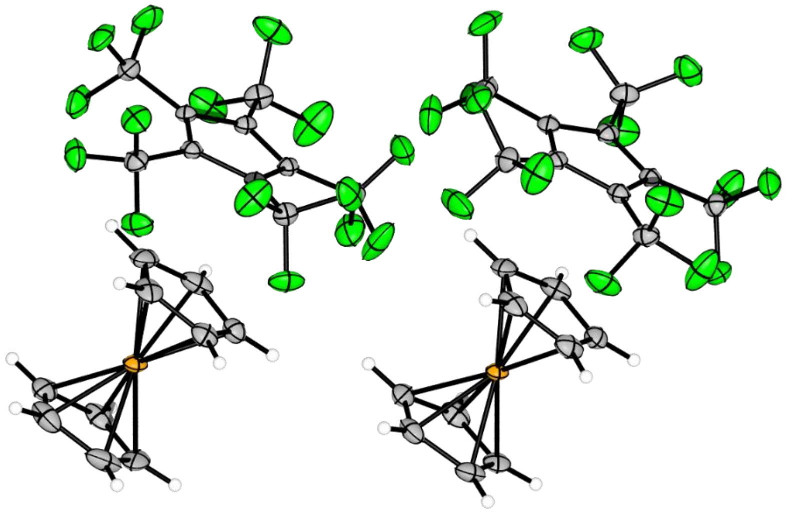

Figure S25. Molecular structure in solid state of [Fe(C_5H_5)$_2$][$C_5(CF_3)_5$] (**5**). Ellipsoids are depicted with 50% probability level. Color code: white-hydrogen, grey-carbon, green-fluorine, orange-iron.

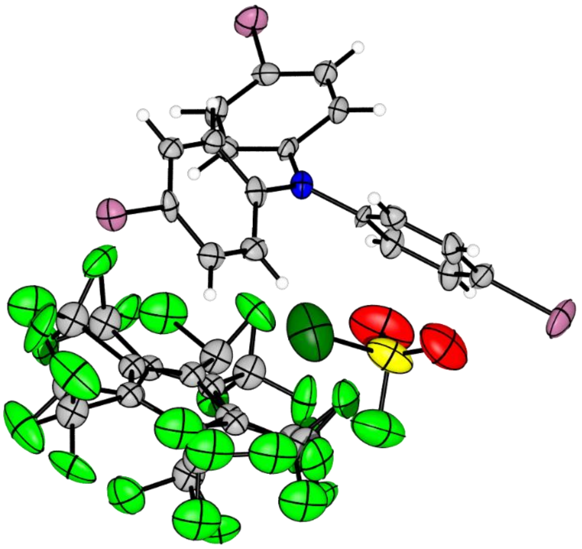

Figure S26. Molecular structure in solid state of [N(*p*-$C_6H_4Br)_3$][$C_5(CF_3)_5$] (**6**) · SO_2ClF. Ellipsoids are depicted with 50% probability level. Color code: white-hydrogen, grey-carbon, green-fluorine, deep green-chlorine, red-oxygen, yellow-sulfur, deep blue-nitrogen, purple-bromine.

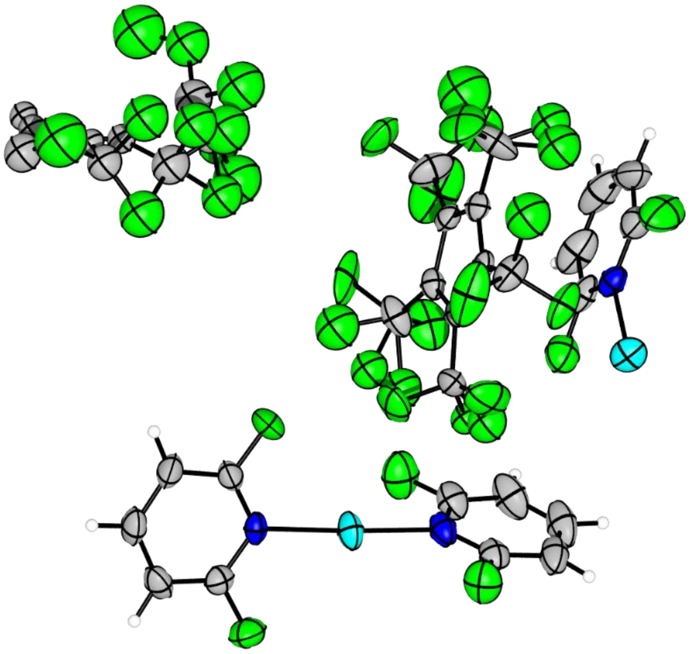

Figure S27. Molecular structure in solid state of [Ag(*o*,*o*-$NC_5H_3F_2)_2$][$C_5(CF_3)_5$] (**7**). Ellipsoids are depicted with 50% probability level. Color code: white-hydrogen, grey-carbon, green-fluorine, light blue-silver, deep blue-nitrogen.

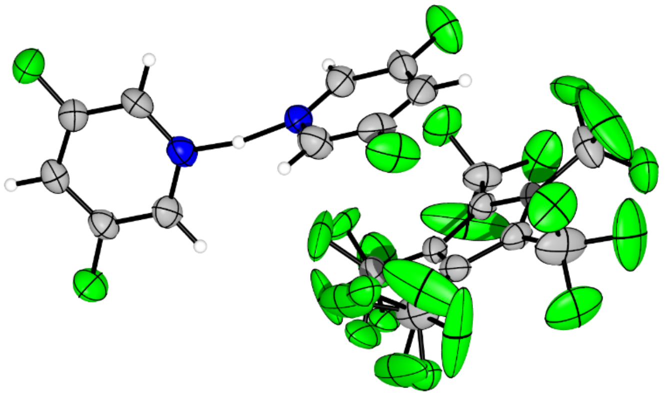

Figure S28. Molecular structure in solid state of [H(*m*,*m*-$NC_5H_3F_2)_2$][$C_5(CF_3)_5$] (**8**). Ellipsoids are depicted with 50% probability level. Color code: white-hydrogen, grey-carbon, green-fluorine, deep blue-nitrogen.

Density functional theory

$[C_5(CF_3)_5]^-$

9	2.210619000	2.648462000	-0.144780000
9	2.987929000	1.032037000	-1.295229000
9	3.218653000	1.036193000	0.849625000
9	0.180482000	3.147165000	1.259813000
9	-1.816571000	2.812056000	0.600897000
9	-0.321652000	3.413251000	-0.820825000
9	-2.899811000	1.314762000	-1.115973000
9	-3.364406000	0.550719000	0.846215000
9	-3.172128000	-0.780628000	-0.830872000
9	-0.329502000	-3.301230000	0.768001000
9	-1.510804000	-2.971232000	-0.993860000
9	-2.232274000	-2.348238000	0.940304000
9	3.149637000	-1.356293000	-0.400883000
9	1.683128000	-2.845898000	-0.858493000
9	2.173406000	-2.394204000	1.193542000
6	-0.223429000	1.182796000	0.019583000
6	-1.173886000	0.149914000	-0.018299000
6	1.054161000	0.595924000	-0.010615000
6	0.888467000	-0.800519000	-0.004091000
6	-0.488245000	-1.072826000	0.016816000
6	2.347942000	1.316890000	-0.141514000
6	-0.536637000	2.619702000	0.247210000
6	-2.633454000	0.309453000	-0.259474000
6	-1.129136000	-2.405707000	0.169023000
6	1.959160000	-1.831012000	-0.014863000

$HC_5(CF_3)_5$

9	3.204507000	-0.206238000	-1.355481000
9	-0.529225000	-3.577591000	0.072698000
9	-3.151031000	-0.232886000	-1.583211000
9	-0.868156000	3.179733000	-0.558340000
9	2.896606000	1.600008000	0.615829000
9	1.854893000	2.713513000	-0.903849000
9	1.205732000	2.801423000	1.152052000
9	3.247194000	-1.121304000	0.594172000
9	2.422708000	-2.188073000	-1.085842000
9	-1.003543000	-2.146851000	1.613099000
9	1.037968000	-2.658428000	1.209909000

9	-1.505137000	2.549140000	1.406387000
9	-2.705490000	2.099017000	-0.315895000
9	-3.392392000	-0.378208000	0.557150000
9	-2.683958000	-2.064940000	-0.574434000
6	1.696091000	1.998641000	0.208452000
6	2.525168000	-0.982598000	-0.509911000
6	-0.143961000	-2.439751000	0.636577000
6	-1.457065000	2.195382000	0.123349000
6	-2.623164000	-0.733745000	-0.465510000
6	0.777295000	0.827779000	-0.020369000
6	-0.692636000	0.910865000	-0.052818000
6	-1.191970000	-0.306621000	-0.288958000
6	1.146672000	-0.433868000	-0.258611000
6	-0.069535000	-1.293240000	-0.407355000
1	-0.077450000	-1.771910000	-1.387260000

$[C_5(CH_3)_5]^-$

6	2.671405000	0.308725000	0.004299000
6	1.120091000	-2.444821000	0.004572000
6	-2.343352000	1.319227000	0.003961000
6	0.531979000	2.636150000	0.005304000
6	0.236667000	1.173502000	-0.005411000
6	1.189124000	0.137550000	-0.005522000
6	-1.980169000	-1.819410000	0.005088000
6	0.498250000	-1.088419000	-0.005368000
6	-0.881112000	-0.810183000	-0.005355000
6	-1.042818000	0.587718000	-0.005447000
1	2.085291000	-2.450545000	-0.506656000
1	1.305938000	-2.841265000	1.011268000
1	0.491245000	-3.181395000	-0.500316000
1	-1.684043000	-2.744348000	-0.494516000
1	-2.308770000	-2.108261000	1.011944000
1	-2.870486000	-1.453088000	-0.510930000
1	-2.730070000	1.525798000	1.010419000
1	-2.265988000	2.287137000	-0.496018000
1	-3.126008000	0.759007000	-0.512507000
1	-0.248849000	3.209685000	-0.498886000
1	0.622557000	3.063889000	1.012260000
1	1.470035000	2.863010000	-0.506142000
1	2.974846000	1.229822000	-0.498072000

1	3.107269000	0.352479000	1.010926000
1	3.176641000	-0.512161000	-0.509495000

$HC_5(CH_3)_5$

6	0.000003000	-2.388159000	0.708600000
6	2.577113000	-0.840051000	-0.265693000
6	1.528999000	2.154536000	0.171990000
6	-1.529002000	2.154534000	0.171987000
6	-2.577112000	-0.840055000	-0.265691000
6	0.736278000	0.908655000	-0.007685000
6	-0.736280000	0.908653000	-0.007684000
6	-1.180218000	-0.343335000	-0.192609000
6	1.180218000	-0.343333000	-0.192610000
6	0.000001000	-1.262175000	-0.321108000
1	0.000002000	-1.975114000	1.714894000
1	-0.878442000	-3.019530000	0.599442000
1	0.878449000	-3.019528000	0.599441000
1	0.000001000	-1.710781000	-1.320923000
1	3.299121000	-0.034969000	-0.170729000
1	2.785475000	-1.563213000	0.523932000
1	2.770419000	-1.347615000	-1.211259000
1	1.300468000	2.631985000	1.124647000
1	2.596953000	1.965070000	0.143100000
1	1.297860000	2.883420000	-0.604607000
1	-1.300451000	2.632001000	1.124631000
1	-1.297885000	2.883404000	-0.604629000
1	-2.596956000	1.965063000	0.143126000
1	-3.299120000	-0.034976000	-0.170711000
1	-2.770423000	-1.347605000	-1.211264000
1	-2.785467000	-1.563230000	0.523924000

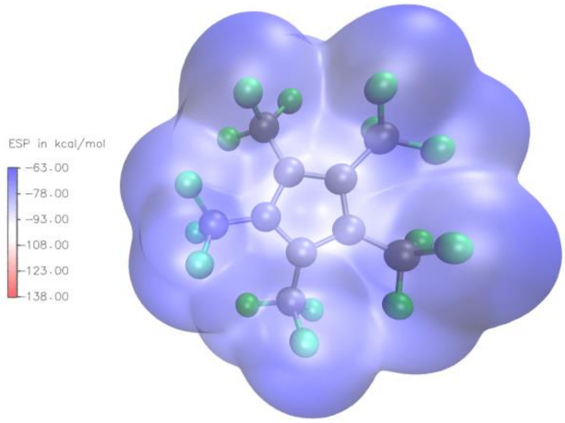

Figure S29. Electrostatic potential of $[C_5(CF_3)_5]^-$ with an isodensity surface of 0.001 e/bohr^3.

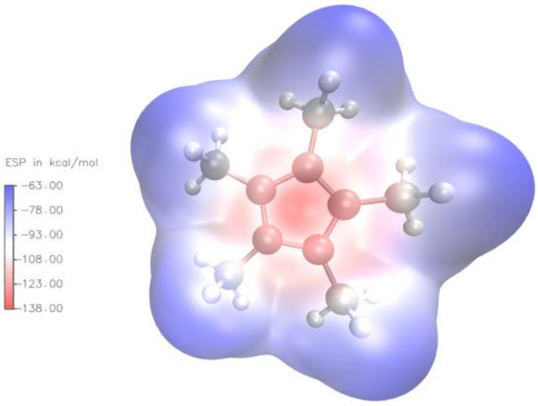

Figure S30. Electrostatic potential of $[C_5(CH_3)_5]^-$ with an isodensity surface of 0.001 e/bohr^3.

References

[73] H. E. Gottlieb, V. Kotlyar, A. Nudelman, *J. Org. Chem.* **1997**, *62*, 7512.

[74] R. K. Harris, E. D. Becker, S. M. Cabral de Menezes, R. Goodfellow, P. Granger, *Pure Appl. Chem.* 2001, *73*, 1795–1818.

[75] M. R. Willcott, *J. Am. Chem. Soc.*, 2009, **131**, 13180.

[76] O. V. Dolomanov, L. J. Bourhis, R. J. Gildea, J. A. K. Howard, H. Puschmann, *J. Appl. Cryst.*, 2009, **42**, 339.

[77] G. M. Sheldrick, *Acta Cryst*., 2015, **A71**, 3–8.

[78] G. M. Sheldrick, *SHELXL Version 2014/7, Program for Crystal Structure Solution and Refinement*; Göttingen, Germany, 2014.

[79] G. M. Sheldrick, *Acta Cryst.*, 2008, **A64**, 112.

[80] K. Brandenburg, Diamond: Crystal and Molecular Structure Visualization http://www.crystalimpact.com/diamond.

[81] Persistence of Vision Pty. Ltd. Persistence of Vision Raytracer. Ltd., Persistence of Vision Pty. 2004.

[82] P. Hohenberg, W. Kohn*, Phys. Rev.* **1964**, *136*, B864

[83] W. Kohn, L. J. Sham*, Phys. Rev.* **1965**, *140*, A1133.

[84] Gaussian 16, Revision C.02, M. J. Frisch, G. W. Trucks, H. B. Schlegel, G. E. Scuseria, M. A. Robb, J. R. Cheeseman, G. Scalmani, V. Barone, G. A. Petersson, H. Nakatsuji, X. Li, M. Caricato, A. V. Marenich, J. Bloino, B. G. Janesko, R. Gomperts, B. Mennucci, H. P. Hratchian, J. V. Ortiz, A. F. Izmaylov, J. L. Sonnenberg, D. Williams-Young, F. Ding, F. Lipparini, F. Egidi, J. Goings, B. Peng, A. Petrone, T. Henderson, D. Ranasinghe, V. G. Zakrzewski, J. Gao, N. Rega, G. Zheng, W. Liang, M. Hada, M. Ehara, K. Toyota, R. Fukuda, J. Hasegawa, M. Ishida, T. Nakajima, Y. Honda, O. Kitao, H. Nakai, T. Vreven, K. Throssell, J. A. Montgomery, Jr., J. E. Peralta, F. Ogliaro, M. J. Bearpark, J. J. Heyd, E. N. Brothers, K. N. Kudin, V. N. Staroverov, T. A. Keith, R. Kobayashi, J. Normand, K. Raghavachari, A. P. Rendell, J. C. Burant, S. S. Iyengar, J. Tomasi, M. Cossi, J. M. Millam, M. Klene, C. Adamo, R. Cammi, J. W. Ochterski, R. L. Martin, K. Morokuma, O. Farkas, J. B. Foresman, and D. J. Fox, Gaussian, Inc., Wallingford CT, 2019.

[85] Y. Zhao, D. G. Truhlar, *J. Phys. Chem.* **2005**, *109*, 5656.

[86] F. Weigend, R. Ahlrichs, *Phys. Chem. Chem. Phys.* **2005**, *7*, 3297.

[87] T. Lu, F. Chen, *J. Comput. Chem.* **2012**, *33*, 580.

[88] J. Zhang, T. Lu, *Phys. Chem. Chem. Phys.* **2021**, *23*, 20323.

[89] W. Humphrey, A. Dalke, K. J. Schulten, *Molec. Graphics* **1996**, *14.1*, 33.

[90] E. P. Janulis, A. J. Arduengo, *J. Am. Chem. Soc.*, 1983, **105**, 3563.

[91] R. D. Chambers, W. K. Gray, J. F. S. Vaughan, S. R. Korn, M. Médebielle, A. S. Batsanov, C. W. Lehmann, J. A. K. Howard, *Perkin Trans.*, **1997**, 135.

[92] R. Sievers, M. Sellin, S. M. Rupf, M. Malischewski, *Angew. Chem. Int. Ed.*, **2022**, *e202211147*.

[93] Trace impurities: 2.98 and 1.21 ppm (1 mol% [NEt_4][$C_5(CF_3)_5$]), 0.08 ppm (H-grease).

[94] [$C_5(CF_3)_5$]$^-$ resonances are supressed and invisible in ^{13}C NMR spectroscopy even at prolonged measurement times.

[95] Trace impurities: 2.08 (Acetone from CD_2Cl_2).

[96] Trace impurities: 2.08 (Acetone from CD_2Cl_2), 1.26 and 0.88 ppm (*n*-pentane from CD_2Cl_2), 0.08 ppm (H-grease).

[97] Trace impurities: 1.26 and 0.88 ppm (*n*-pentane from CD_2Cl_2).

[98] The high R-values originate from pronounced disordering of the CF_3-groups around the C-C axis.
